# Involvement of human ribosomal proteins in nucleolar structure and p53-dependent nucleolar stress

**DOI:** 10.1038/ncomms11390

**Published:** 2016-06-06

**Authors:** Emilien Nicolas, Pascaline Parisot, Celina Pinto-Monteiro, Roxane de Walque, Christophe De Vleeschouwer, Denis L. J. Lafontaine

**Affiliations:** 1 RNA Molecular Biology, F.R.S./FNRS, Université Libre de Bruxelles, B-6041 Charleroi-Gosselies, Belgium; 2 Center for Microscopy and Molecular Imaging, B-6041 Charleroi-Gosselies, Belgium; 3 ICTEAM-ELEN, Université catholique de Louvain, B-1348 Louvain-la-Neuve, Belgium

## Abstract

The nucleolus is a potent disease biomarker and a target in cancer therapy. Ribosome biogenesis is initiated in the nucleolus where most ribosomal (r-) proteins assemble onto precursor rRNAs. Here we systematically investigate how depletion of each of the 80 human r-proteins affects nucleolar structure, pre-rRNA processing, mature rRNA accumulation and p53 steady-state level. We developed an image-processing programme for qualitative and quantitative discrimination of normal from altered nucleolar morphology. Remarkably, we find that uL5 (formerly RPL11) and uL18 (RPL5) are the strongest contributors to nucleolar integrity. Together with the 5S rRNA, they form the late-assembling central protuberance on mature 60S subunits, and act as an Hdm2 trap and p53 stabilizer. Other major contributors to p53 homeostasis are also strictly late-assembling large subunit r-proteins essential to nucleolar structure. The identification of the r-proteins that specifically contribute to maintaining nucleolar structure and p53 steady-state level provides insights into fundamental aspects of cell and cancer biology.

## Abstract (web summary)

The nucleolus is a specialized functional domain of the nucleus where ribosome biogenesis is initiated and also implicated in a p53-dependent anti-tumor surveillance. Here the authors use a quantitative imaging approach to detail the role of each ribosomal protein on the structural integrity of the nucleolus and p53 homeostasis.

## Graphical abstract

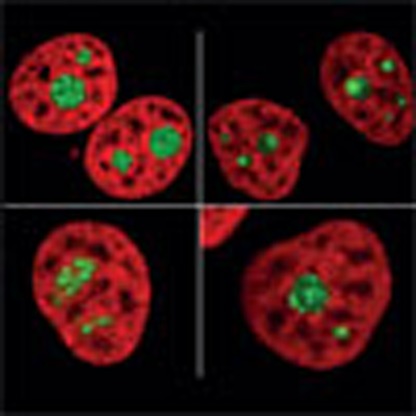

Within the nucleus, the nucleolus is a specialized functional domain essential to gene expression^1^. It is the site where the initial steps of ribosome biogenesis take place^2^. Ribosomes are ribonucleoprotein nanomachines converting the genetic information encoded in messenger RNAs (mRNAs) into proteins. The human ribosome contains four ribosomal RNA (rRNAs) and 80 r-proteins organized in two subunits, each performing specialized functions in translation^3^,^4^. The small subunit (SSU), which consists of a single rRNA (18S) and 33 r-proteins, decodes the mRNA, while the large subunit (LSU), comprising three rRNAs (5S, 5.8S and 28S) and 47 r-proteins, bears the peptidyl transferase centre where amino acids are joined together into proteins. In the nucleolus, the 18S, 5.8S and 28S rRNAs are synthesized by RNA polymerase I (Pol I) as long precursors, pre-rRNAs are modified, folded and processed, and most r-proteins are assembled to form ribosomal subunits^2^.

r-proteins are not involved in ribosome-mediated catalysis of peptide bond formation^3^,^5^. Nonetheless, r-proteins play essential roles in shaping and maintaining the overall structure of the ribosomal subunits, and mutations in r-proteins are frequently associated with developmental disorders and human diseases^6^. Notably, ribosomopathies are cancer predisposition syndromes caused by ribosome biogenesis dysfunction^7^, due to mutations in r-proteins or ribosomal assembly factors. r-proteins are intimately linked to tumourigenesis, being directly involved in regulating the steady-state level of the anti-tumor protein p53 (ref. 8). This occurs via activation of specific anti-tumor surveillance pathways, through direct binding of specific r-proteins to the p53 regulator Hdm2 (see below and ref. 9).

The nucleolus is not limited by a lipid membrane. This makes it a highly dynamic structure that responds promptly, sometimes by profound morphological and compositional alterations, to cell stresses such as viral infections, DNA damage and drug treatments^10^,^11^. During interphase, the nucleoli of amniotic eukaryotes display three morphologically distinct layers^12^,^13^, which can be drastically re-organized under stress^14^. During mitosis, the nucleolus undergoes a dramatic cycle of disassembly/reassembly that parallels Pol I activity controlled by specific phosphorylations^15^,^16^. The number of nucleoli per cell nucleus and the shape and size of the nucleoli also vary greatly in proliferative diseases such as cancers^17^. Cancer cells are more sensitive than non-cancer cells to inhibition of ribosome synthesis, and are killed selectively by treatment with Pol I inhibitors^18^,^19^. Despite the importance of the nucleolus as a cell stress sensor^20^, disease biomarker and target for cancer therapy^21^, how its structural integrity is maintained remains totally unclear.

While the principles of assembly and maintenance of the nucleolus are far from being understood^14^, r-proteins which are very abundant, very basic and which assemble mostly in the nucleolus onto pre-rRNAs to form ribosomal subunit precursors are likely to play an important role. The assembly of r-proteins is not random but follows a precise sequence of events. Groups of r-proteins have been defined on the basis of their assembly at early, intermediate or late stages of ribosomal subunit biogenesis^22^. Compromising the timely association of r-proteins with rRNA can indeed lead to severe pre-rRNA processing inhibitions, ribosomal subunit synthesis abortion and sometimes to nucleolar structural alterations visible at the microscopic level^23^. To date, no attempt has been made to systematically address the involvement of r-proteins in nucleolar structure maintenance or to grade their involvement in this process. Here we have depleted human cells systematically of each of the 80 r-proteins and investigated the consequences on nucleolar structural integrity, pre-rRNA processing, accumulation of mature rRNAs and p53 steady-state level (see experimental strategy in Fig. 1a).

## Results

### Effects of r-protein depletion on nucleolar structure

Human cells stably producing the nucleolar methyltransferase fibrillarin (FBL) fused to a green fluorescent protein (GFP) were transfected with siRNAs targeting the appropriate transcripts, incubated for 3 days, and imaged by fluorescence microscopy (Methods section). Each r-protein was depleted in three experiments, a different siRNA being used in each experiment. The entire screen was duplicated. A non-targeting siRNA (SCR), mock-treated cells (MOCK) and a calibration set were included (Methods section and Supplementary Fig. 1). The calibration set consisted of proteins whose depletion leads to moderate to severe nucleolar disruption, formation of nucleolar ‘caps' (see below), or a reduction in fluorescence intensity (Supplementary Fig. 1).

**Figure 1:**
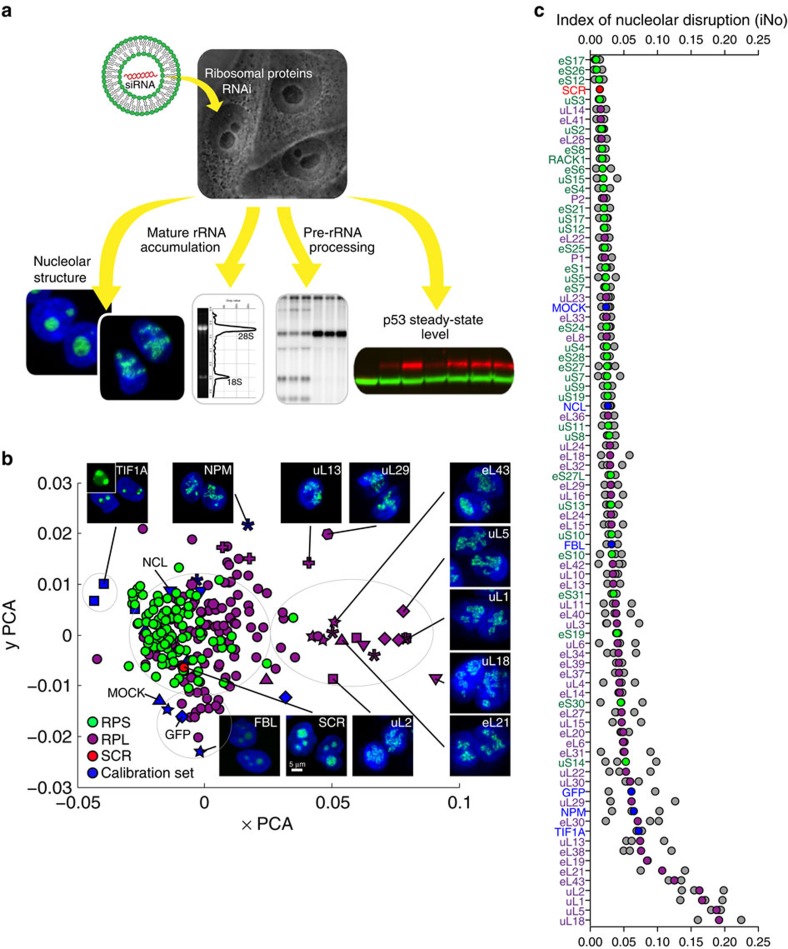
Systematic screening of human r-proteins reveals that uL5 (RPL11) and uL18 (RPL5) are the strongest contributors to nucleolar structure maintenance. (**a**) Experimental strategy: all 80 r-proteins were depleted one by one in human cells by use of specific siRNAs. The nucleolar structure (fluorescence microscopy), the accumulation of mature 18S and 28S rRNAs (electropherograms), pre-rRNA processing (high-resolution northern blotting), and steady-state accumulation of p53 (fluorescent western blotting) were monitored. (**b**) PCA showing a classification of r-proteins according to their requirement for nucleolar structure maintenance. Each r-protein was depleted in three knockdown experiments, each performed with a different siRNA. The image-processing algorithm that we designed for this analysis involves selecting five discriminant shape and textural features, computing five *d*_k_ values, and reducing the five dimensions to two by PCA. In the resulting plot, each coloured dot represents one population of cells treated with one siRNA. Dot colour is indicative of the targeted protein: green for SSU r-proteins and magenta for LSU r-proteins. The mean of three populations of cells treated with a non-targeting control siRNA (SCR) is shown in red. Blue symbols represent the six calibration controls (FBL, GFP, nucleolin, nucleophosmin, MOCK and TIF1A, see Supplementary Fig. 1). Insets show images of the nuclei of cells depleted of representative proteins with the DNA stained in blue and the nucleoli appearing in green (FBL). For a few representative examples, a specific symbol is used (for example, a diamond for uL5). RPL, r-proteins of the LSU; RPS, r-proteins of the SSU. (**c**) r-proteins and calibration controls classified according to the severity of nucleolar disruption caused by their absence. The iNo was defined as the sum of the *d*_k_ values of the five most discriminant shape and textural features identified in this work (Methods section). Higher iNo correspond to more severe disruption. Colour-coding as in **b**. The coloured dots are the means of three individual experiments (shown in grey). Note: the r-proteins are named according to a recently revised nomenclature^24^ where the ‘e' prefix stands for eukaryote-specific and ‘u' for universal (present in bacteria, archaea and eukaryotes).

To characterize nucleolar morphology defects both qualitatively and quantitatively, we developed a specific image-processing algorithm. Briefly, we first segmented the observed nuclei on the basis of shape- and size-consistent adaptive thresholding of a nuclear stain (4,6-diamidino-2-phenylindole (DAPI) signal). Then, within each nuclear mass, GFP signal thresholding and mathematical morphology (Methods section) were applied to segment nucleoli into connected components. To optimize discrimination of nucleoli of cells depleted of an r-protein from those of SCR-treated control cells, five shape and textural features were extracted from the largest connected components of each nucleolus. These five features, selected from a set of 11 as the most discriminant ones, were: area, elliptical regularity, percentage of pixels below an optimized intensity threshold, smallest intensity and number of local minima (Methods section). For each of the five features, a *d*_k_ value corresponding to a statistically significant distance between the feature distribution in cells depleted of an r-protein and control cells were computed. Each population of cells was thus characterized by five *d*_k_ values. Principal component analysis (PCA) was used to reduce these five dimensions to two, allowing ready visualization of the data in a scatter plot (Fig. 1b) where each dot corresponds to a population of cells treated with one siRNA.

The PCA revealed groups of proteins whose depletion leads to similar nucleolar morphological phenotypes (Fig. 1b). Four major groups emerged. The largest one, indicated by a grey ellipse containing the SCR control (shown as a red dot), comprises r-proteins whose depletion had no significant impact on nucleolar structure. Importantly, most of the r-proteins are in this group, that is, nearly all of the SSU proteins (shown in green) and roughly two-thirds of the LSU proteins (in magenta). A second group, beneath the SCR control, comprises proteins whose depletion did not alter the nucleolar structure but reduced the fluorescence intensity (for example, control cells treated with siRNAs against GFP or FBL, see also Supplementary Fig. 1). Cells depleted of the RNA Pol I transcription factor TIF1A formed distinctive ‘nucleolar caps', in keeping with the known effects of RNA Pol I inhibitions^14^, and appeared isolated in the upper left part of the graph. The fourth group comprises the few r-proteins whose depletion was found to impact nucleolar structure very severely, remarkably they are almost exclusively LSU proteins. This cluster forms a tail in the right part of the graph. In cells depleted of these major contributors to normal nucleolar structure, the nucleoli were detected as ‘unfolded beaded necklaces' (Fig. 1b). Our automated classification was benchmarked with a manual one and found to be extremely robust (Supplementary Fig. 2). The complete data set is available in an information-rich database at http://www.RibosomalProteins.com.

To stratify the r-proteins according to the severity of nucleolar disruption caused by their absence, we defined an index of nucleolar disruption (iNo) as the sum of the absolute values of the five *d*_k_ distances (Methods section). For each r-protein, an average iNo, based on the values obtained with the three different siRNAs used, was calculated and plotted. In the resulting graph, the r-proteins are listed from top to bottom in the order of increasing impact on nucleolar structure (Fig. 1c). As concluded from the PCA, depletion of most r-proteins appears to have no significant impact on nucleolar structure (iNo<0.05), and the proteins whose depletion has the greatest effect belong to the LSU (magenta). Unexpectedly, the r-proteins uL5 (formerly RPL11, ref. 24) and uL18 (formerly RPL5) appear among the strongest contributors to maintenance of nucleolar structural integrity (Fig. 1c). These are precisely the proteins which, together with the 5S rRNA, form a small ribonucleoprotein complex, the 5S ribonucleoprotein, which acts as an HDM2 trap and controls the steady-state level of p53 in a regulatory circuit known as p53-dependent anti-tumor nucleolar surveillance^25^,^26^. Briefly, in unstressed cells, p53 is constitutively targeted for proteosomal degradation by Hdm2-mediated ubiquitination. In the event of a nucleolar stress, such as a ribosome biogenesis dysfunction, unassembled ribosomal components accumulate. These include the 5S ribonucleoprotein, which interacts with Hdm2, sequestering it away from p53. As a result, p53 is stabilized and induces cell cycle arrest and cell death^9^. In mature 60S subunits, the 5S ribonucleoprotein constitutes the central protuberance (CP), a late-assembling structure (see below).

**Figure 2:**
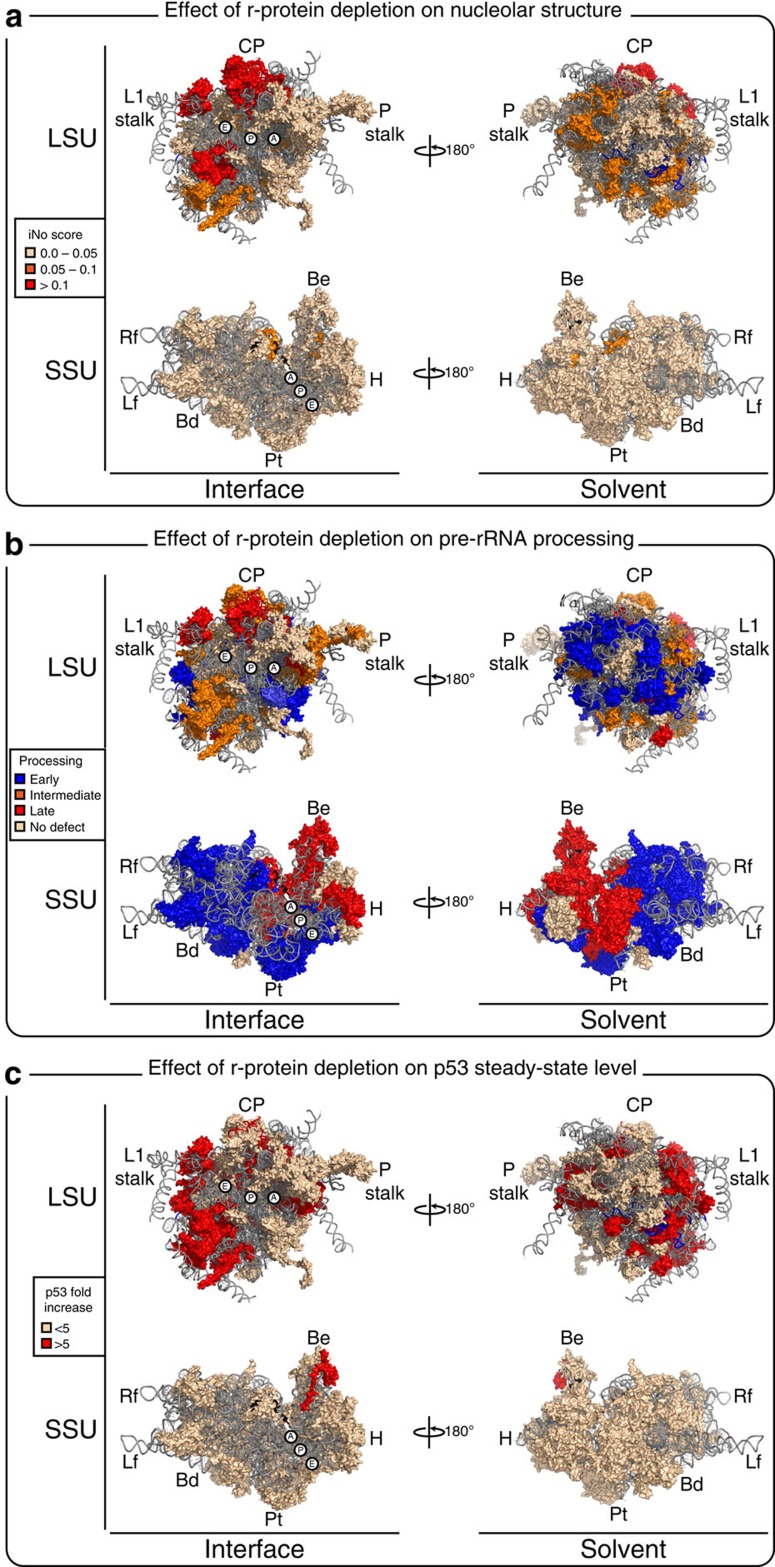
Late-assembling r-proteins of the LSU are the strongest contributors to nucleolar structure maintenance and p53 homeostasis. Three-dimensional (3-D) models of human ribosomal subunits based on protein data bank (PDB) entries 3J3D, 3J3A, 3J3F and 3J3B. The r-proteins are colour-coded according to the impact of their depletion on nucleolar structure (iNo values) (**a**), pre-rRNA processing (**b**) or the p53 steady-state level (**c**). Left, subunit interface views; right, solvent-exposed views. The aminoacyl (A), peptidyl (P) and exit (E) transfer RNA (tRNA) sites are indicated. Morphological features of the subunits are highlighted. On the LSU: the L1-stalk, CP and phospho-stalk (P-stalk). On the SSU, the beak (Be), head (H), platform (Pt), body (Bd), left foot (Lf) and right foot (Rf).

Ribosomal subunit assembly is a sequential process involving progressive binding of r-proteins to nascent rRNAs and gradual formation of ribosomal landmarks^23^,^27^,^28^,^29^,^30^,^31^. We wondered if the r-proteins important for nucleolar structure might map to particular areas on mature ribosomal subunits. Colour-coding of the r-proteins according to their iNo values, on a three-dimensional model based on the crystal structure of the human ribosome^32^ (Fig. 2a), made it obvious that the strongest contributors to nucleolar structure maintenance belong to the LSU and are not randomly distributed over it: rather, they are preferentially located at the subunit interface in areas corresponding to the CP, the L1-stalk and a region directly below the L1-stalk (Fig. 2a). All of these are late-forming structures (see below).

The nucleolus is a highly dynamic structure capable of responding through profound morphological alterations to cellular stresses such as drug treatment or viral infection^20^. In interphase, however, it is quite stable. It is disassembled at the onset of mitosis and reassembled at the end of this process^14^. In our nucleolar screens, cells were imaged after 3 days of r-protein depletion, as we reasoned that cells might have to undergo at least two cycles of nucleolar breakdown/nucleolar genesis for nucleolar alterations to become readily detectable. This assumption was confirmed when we established the time course of the appearance of nucleolar morphological defects (Supplementary Fig. 3). Focusing on 13 representative r-proteins, and monitoring changes at 24-h intervals over a 3-day depletion period, we indeed found nucleolar disruption to increase steadily (Supplementary Fig. 3b), in parallel with an increase in iNo values. Nucleolar disruption became obvious only after 72 h of depletion (Supplementary Fig. 3a).

The nucleoli of cells of amniotic organisms have three nucleolar subcompartments^12^,^13^,^33^. In our original screens, we used FBL, a dense fibrillar component marker, to assess nucleolar morphology. To extend our conclusions, we examined whether nucleolar structural defects due to r-protein depletion might be equally observable with a marker of a different nucleolar subcompartment. We chose to monitor by immunofluorescence a granular component marker, the PES1 antigen, in depletion experiments focusing on 13 representative r-proteins (Supplementary Fig. 4). As expected for a granular component protein, PES1 staining was peripheral to the FBL signal (Supplementary Fig. 4b). Remarkably, we observed extreme closeness between the iNo scores computed from the FBL and PES1 signals, and the ranking of r-proteins according to phenotype severity was largely similar (Supplementary Fig. 4a). We conclude that the nucleolar structural defects due to r-protein depletion can be monitored similarly with a dense fibrillar component or a granular component antigen.

**Figure 3:**
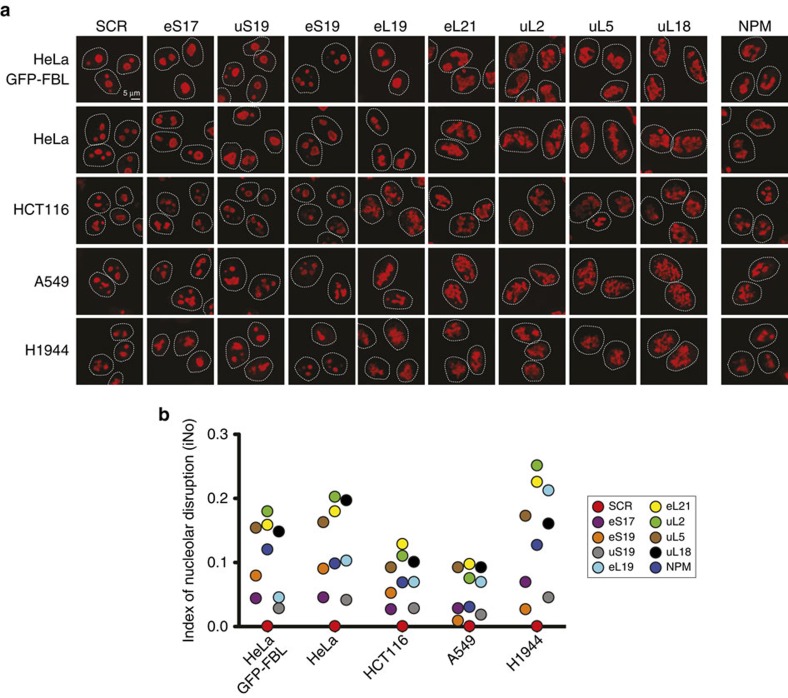
Quantitative monitoring of nucleolar morphology in different human cell lines based on detection of endogenous PES1. The data show, for a selection of eight representative r-proteins, that the r-proteins contributing weakly or strongly to nucleolar structure maintenance are largely the same in multiple cell lines. (**a**) The indicated r-proteins were depleted with an siRNA for 3 days in two cervical carcinoma cell lines (HeLa-GFP-FBL, engineered to express green fluorescent FBL, and genetically unmodified HeLa), one colon carcinoma cell line (HCT116) and two lung carcinoma cell lines (A549 and H1944). Endogenous PES1 was detected by immunostaining with a specific antibody (Methods section). As a control, cells were treated with a non-targeting control siRNA (SCR) and depleted of nucleophosmin (NPM; Supplementary Fig. 1). (**b**) Values of the nucleolar disruption index (iNo) obtained after 3 days of siRNA-mediated depletion of the indicated r-protein as calculated on the basis of the endogenous PES1 signal.

We conducted our nucleolar screens in HeLa cells because of the large size of their nucleus, which makes them ideal for use in high-throughput screens with visual readouts (for example, refs 34, 35). To see how general the effects observed in HeLa-GFP-FBL cells might be, we tested five cell lines: two cervical carcinoma cell lines (HeLa-GFP-FBL and HeLa), one colon carcinoma cell line (HCT116) and two lung cancer cell lines (A549 and H1944). We selected eight representative r-proteins, depleted them for 3 days in each of the five cell lines and monitored nucleolar structure by immunostaining of endogenous PES1 and iNo score computation (Fig. 3). For the r-proteins tested, we found the weak and strong contributors to nucleolar structure maintenance to be largely the same in all five cell lines (Fig. 3), with uL5 and uL18 playing an important role in each case.

### Effects of r-protein depletion on pre-rRNA processing

**Figure 4:**
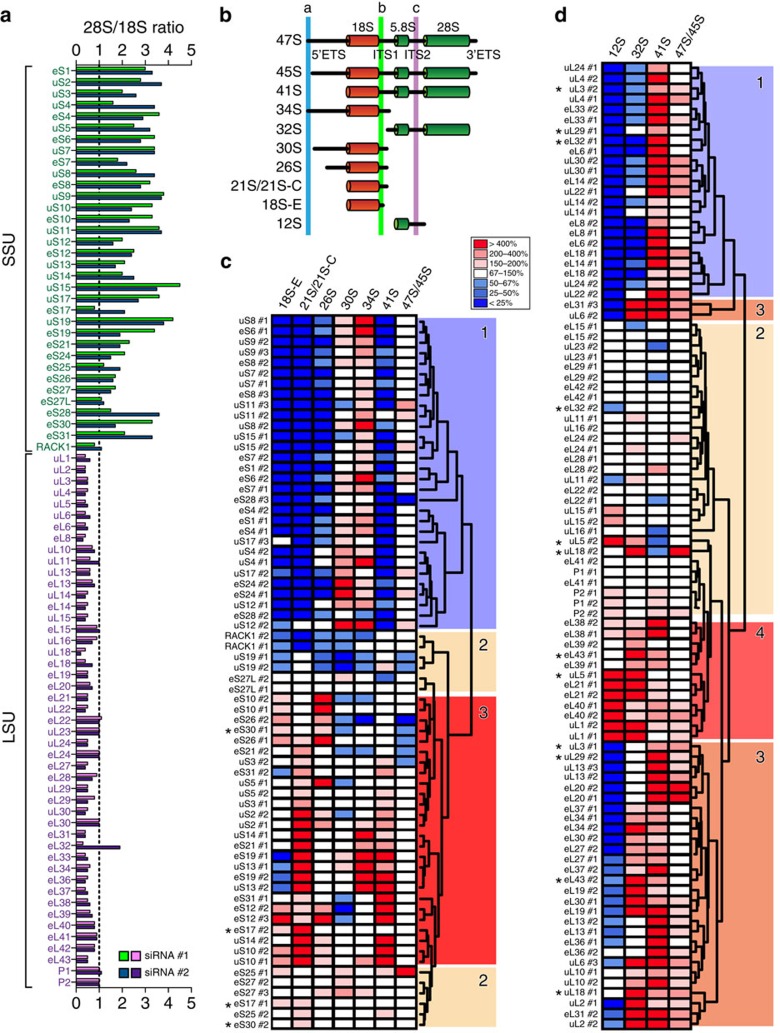
Involvement of human r-proteins in pre-rRNA processing. (**a**) The 28S/18S ratio calculated from Agilent bioanalyzer electropherograms. Data are shown for the two different siRNAs used (siRNA #1 and #2). (**b**) Major pre-rRNA intermediates and probes used in this work. Three of the four rRNAs are produced by RNA Pol I as a long 47S primary transcript. The 18S, 5.8S and 28S rRNAs are separated by noncoding external (ETS) and internal (ITS) transcribed spacers. Probes a, b and c are the oligonucleotides LD1844, LD1827 and LD1828, respectively (Methods section). (**c**) Pre-rRNA processing inhibitions after depletion of SSU r-proteins. On the northern blots (www.RibosomalProteins.com, Supplementary Figs 5,6 and 11), all RNA species were quantified with a Phosphorimager, normalized with respect to the non-targeting control (SCR), and their abundances represented on a heatmap using the colour code indicated. The heatmap profiles were clustered with ‘R' and the corresponding proteins grouped in classes of r-proteins affecting the same or similar processing steps. The different siRNAs used are indicated (#). Asterisks (*) refer to r-proteins assigned to two groups according to the siRNA used. (**d**) As in **c** for LSU r-proteins.

In an attempt to correlate the effects of r-protein depletion on nucleolar structure with defects in ribosome biogenesis, we determined which r-proteins are essential to pre-rRNA processing (Fig. 4). Mature rRNAs are produced from long precursor molecules. They are embedded in noncoding spacers and require extensive processing to be generated^2^,^36^. Pre-rRNA processing analysis is a good proxy for ribosomal assembly analysis, because failure of an r-protein to bind to nascent ribosomes leads to ribosome biogenesis blockade, pre-rRNA processing inhibitions and subunit biogenesis abortion^23^,^30^,^31^,^37^.

The synthesis of each of the 80 r-proteins was knocked down for 2 days in HCT116 cells with an appropriate siRNA. Total RNA was then extracted, run on a bioanalyzer and analysed by high-resolution quantitative northern blotting. Two different siRNAs were used for each r-protein and yielded largely similar results (Fig. 4). As controls, we used UTP18 and NOL9 because their depletion leads to well-established pre-rRNA processing defects (Supplementary Figs 5,6 and 11, www.RibosomalProteins.com; see ref. 38). As further controls, non-targeting siRNA (SCR) and mock-treated cells were used (Supplementary Figs 5 and 6). HCT116 and HeLa cells are both of epithelial origin and, as shown above, their nucleolar structure is similarly affected by r-protein depletion (Fig. 3). We performed our RNA processing work and p53 steady-state accumulation analysis (see below) on HCT116 cells because, unlike HeLa cells, they express p53 normally^39^. For the RNA analysis, cells were depleted for only 2 days, as we had established beforehand, precisely in HCT116 cells, that *bona fide* pre-rRNA processing inhibitions are early defects preceding cell cycle arrest and apoptosis and are best captured at this time point (discussed in ref. 38).

The ratio of 28S to 18S mature rRNA was extracted from bioanalyzer electropherograms (Fig. 4a). The accumulation of SSU 18S rRNA was strongly decreased, and the 28S/18S ratio accordingly increased, by SSU r-protein depletion (Fig. 4a). Reciprocally, LSU r-protein depletion led to decreased accumulation of the LSU 28S rRNA and to a reduced 28S/18S ratio (Fig. 4a). Northern blots were probed with specific radioactively labelled oligonucleotides, revealing all major known pre-rRNA intermediates (Fig. 4b, Supplementary Figs 5, 6 and 11 and www.RibosomalProteins.com). Each band detected was quantified with a phosphoimager and normalized with respect to the SCR control. The signals were represented on heatmaps (Fig. 4c for SSU r-proteins, Fig. 4d for LSU r-proteins and Supplementary Fig. 11; see also ref. 38). The heatmaps were clustered with the software ‘R', revealing functionally related groups of r-proteins whose depletion affects similar processing steps (Fig. 4c,d, and Supplementary Figs 5, 6 and 11). For the SSU r-proteins, three groups emerged: proteins whose depletion affects early processing (class 1), late processing (class 3) or has no significant effect on processing (class 2; Fig. 4c, see representative examples in Supplementary Fig. 5 and www.RibosomalProteins.com and Supplementary Fig. 11 for a full data set). Our classification of the SSU r-proteins corresponds largely to that previously established in HeLa cells^23^. We identified four classes of LSU r-proteins (Fig. 4d and Supplementary Figs 6 and 11): those whose depletion affects early cleavage steps (class 1), intermediate cleavage steps (class 3), late cleavage steps (class 4) or has no substantial impact on processing (class 2; Fig. 4d and Supplementary Fig. 6). Importantly, no such classification of LSU r-proteins has been reported previously. Our classification of r-proteins' involvement in pre-rRNA processing thus confirms and largely extends previous work (Supplementary Fig. 7).

The r-proteins were mapped on a three-dimensional model of the human ribosome according to their involvement in processing (Fig. 2b). This revealed, on both subunits, a strikingly asymmetric distribution. On the mature SSU, the r-proteins required for early processing steps are those forming the body and platform (Fig. 2b), both of which are known as early-assembling subunit structures^27^,^28^,^40^. The r-proteins affecting late cleavage steps, in contrast, correspond to the head and beak (Fig. 2b), which are late-forming structures^27^,^28^,^40^. On the LSU, the r-proteins important for early processing are mainly exposed on the solvent side of the ribosome (in blue on the right-hand-side cartoon Fig. 2b), while those required for intermediate cleavages are at the interface side (in orange on the left-hand-side cartoon), below the L1 stalk, and those important for late processing correspond largely to the CP and L1-stalk (in red). Remarkably, this is precisely the order in which these structures have been shown to form in budding yeast^30^.

A comparison of our nucleolar structure and rRNA processing data reveals that the r-proteins whose depletion has the greatest effect on nucleolar structure (in red in Fig. 2a) are largely those required for intermediate or late processing steps in the formation of the large ribosomal subunit (in orange and red in Fig. 2b). Within this subunit, they belong mostly to late-assembling structures, including the L1-stalk and CP (uL5 and uL18). In conclusion, while practically all r-proteins appear important for pre-rRNA processing, most of them have no incidence on the structural integrity of the nucleolus.

**Figure 5:**
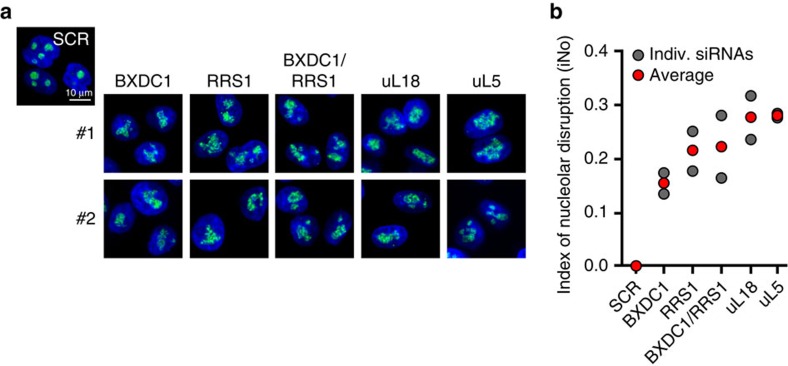
The central protuberance assembly factors BXDC1 and RRS1 are required for nucleolar structure integrity. (**a**) Cells expressing FBL fused to the GFP were treated for 3 days with an siRNA targeting transcripts encoding the indicated protein. Two independent siRNAs (#1 and #2) were used in each case. Cells treated with a non-targeting (SCR) siRNA control are shown for reference. (**b**) For each depletion, the nucleolar disruption index (iNo) was calculated (see Fig. 1 and Methods section).

Several *trans*-acting factors, including BXDC1 and RRS1, are required for CP assembly^26^,^41^. This function is conserved between yeast and human^26^,^41^. Considering the strong effect of uL5 or uL18 depletion on nucleolar structure, and because both of these proteins are CP components, we predicted that depletion of factors involved in CP formation should also cause profound nucleolar structure alterations. This proved to be true: we found depletion of BXDC1, RRS1 or both to affect nucleolar structure severely (Fig. 5a), almost as strongly as does uL5 or uL18 depletion (Fig. 5b).

### Effects of r-protein depletion on p53 steady-state levels

**Figure 6:**
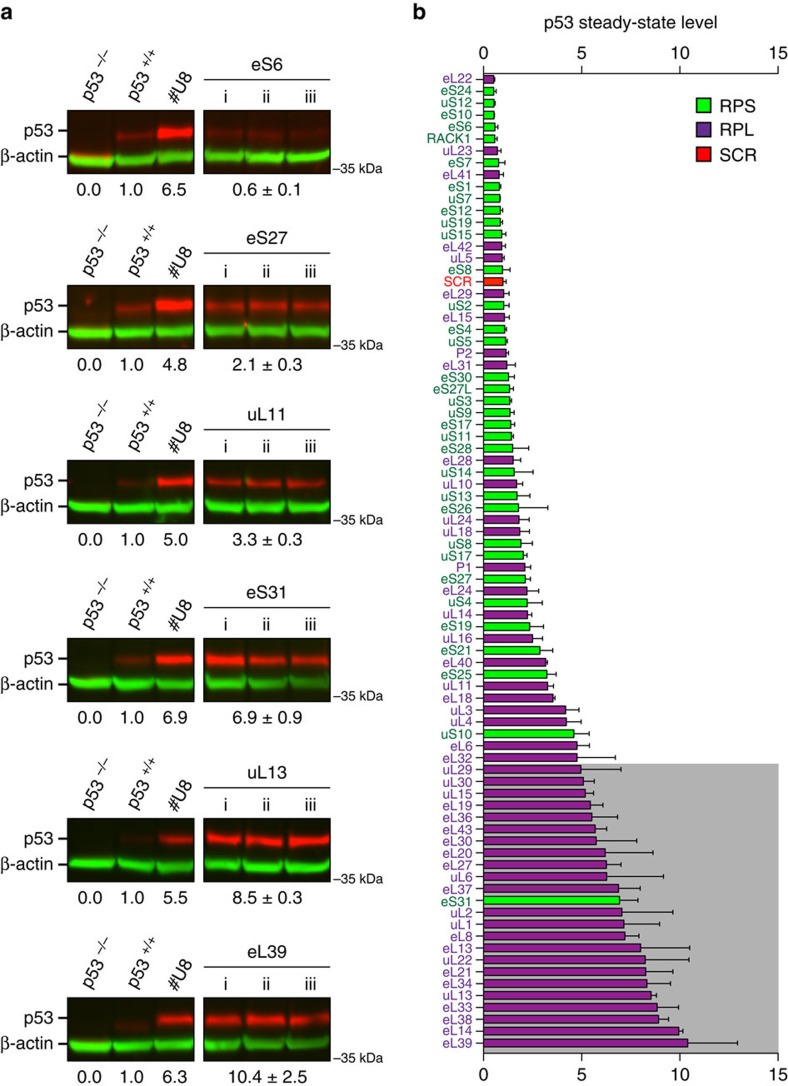
Involvement of human r-proteins in p53 homeostasis. (**a**) Steady-state level of p53 determined by quantitative fluorescent western blotting. Western blots analysis are shown for representative r-proteins, with the p53 level indicated underneath as a mean of biological triplicates obtained after treatment of cells with the same siRNA (i, ii and iii). The siRNA used was selected on the basis of its proven efficacy in the processing and nucleolar screens (Figs 1 and 4). The p53 signal corrected for loading (using β-actin as reference) was expressed with respect to the level observed in cells treated with a non-targeting siRNA control (p53^+/+^). Red signal, p53; green signal, β-actin. A complete data set for all 80 r-proteins is available at www.RibosomalProteins.com and in Supplementary Fig. 11. As loading control we used HCT116 p53^+/+^ cells transfected with a non-targeting siRNA (p53^+/+^) providing the basal level of p53 or with an antisense oligonucleotide suppressing the activity of the box C/D snoRNA U8 (#U8), thereby stimulating p53 accumulation up to sixfold (D.L.J.L. submitted). As background control, we used a matched isogenic HCT116 cell line that does not express p53 (HCT116 p53^−/−^, (ref. 39)) treated with a non-targeting siRNA (p53^−/−^). (**b**) r-proteins classified according to their impact on the p53 steady-state level. The non-targeting (SCR) control is shown in red, the SSU r-proteins in green, and the LSU r-proteins in magenta. The histogram bars are the means of triplicates with s.d. r-proteins whose depletion leads to a fivefold increase in p53 level are highlighted in a grey box.

Given the numerous connections between p53 and ribosomal component synthesis on the one hand^42^, and between the functional integrity of the nucleolus and p53 metabolic stability on the other^43^, we examined systematically how depletion of each individual r-protein might affect the steady-state level of p53. Colon carcinoma cells expressing p53 (HCT116 p53^+/+^, ref. 39) were transfected with one siRNA targeting each r-protein transcript and incubated for 2 days. In this analysis we used a single siRNA, selected on the basis of its proven efficacy in the nucleolar and processing screens, and carried out depletion for 2 days to allow a direct comparison with the RNA analysis. Total protein was then extracted and analysed by quantitative fluorescent western blotting (Fig. 6a and Supplementary Fig. 11).

The p53 steady-state level increase observed ranged from 0 to 10-fold (Fig. 6b). About a third of the r-proteins (24/80) were found to affect p53 level at least fivefold (Fig. 6b, grey box), the cutoff we adopted arbitrarily as significance threshold. As observed for the effects on nucleolar structure, we found nearly all of these r-proteins to belong to the LSU, the sole exception being eS31. Interestingly, depletion of uL5 or uL18, involved in p53-dependent nucleolar surveillance (discussed above), had no significant impact on the p53 steady-state level, in keeping with previous reports^25^,^26^,^44^ and in contrast to the role of these proteins in forming an Hdm2 trap when they accumulate in cells^44^,^45^,^46^. As an additional control we established by reverse transcription with quantitative PCR (RT–qPCR), for 48 r-proteins whose depletion did not significantly affect p53 accumulation (see Fig. 6b, from eL22 to eS21), the efficiency of r-protein depletion at the mRNA level (Supplementary Fig. 8). We found depletion to be effective for all the r-proteins tested, the residual mRNA level for most of them (40 out of 48) being below 20%. Note that 31 out of the 48 candidates tested showed a marked processing defect on depletion (Fig. 4, Supplementary Figs 5,6 and 11), a further indication that depletion was efficient.

In view of the model of nucleolar stress above, we wondered if the significant increase in p53 observed on depletion of 24 r-proteins might involve uL5 and uL18. We found this to be the case: co-depletion of any one of the 24 r-proteins and either uL5 or uL18 led to normal levels of both p53 and its transcriptional target p21 (Supplementary Fig. 9a). The effects of BXDC1 and RRS1 were also investigated. As expected from the role of these proteins as ribosome (CP) assembly factors, their depletion also caused p53 and p21 to increase, and this rise was dependent on uL5 and uL18 (Supplementary Fig. 9b).

Figure 2c shows the distribution of the r-proteins on mature subunits according to their impact on p53 expression. This shows that the significant contributors to p53 homeostasis all correspond to late-assembling structures on the subunits (Fig. 2b,c).

## Discussion

In summary, we show here that depletion of the vast majority of the 80 human r-proteins does not impact nucleolar structure. This notably applies to nearly all SSU r-proteins (Fig. 1). In striking contrast, about a third of the LSU proteins appear essential to maintaining normal nucleolar structure. This marked dichotomy is in line with the notion that pre-40S subunits are exported to the cytoplasm more rapidly than pre-60S subunits, whose production is more complex and requires numerous additional nuclear maturation steps. Among the strongest contributors to nucleolar structure are uL5 and uL18, known to form with 5S rRNA an Hdm2 trap and p53 stabilizer^25^,^26^.

Most r-proteins assemble with pre-rRNAs within the nucleolus, quite early in the subunit assembly process. Notable exceptions are the acidic proteins uL10 (formerly P0), P1 and P2, which form the P-stalk on pre-60S subunits only after reaching the cytoplasm in yeast^47^. Accordingly, we found the acidic r-proteins to have no impact on nucleolar structure (Supplementary Fig. 11 and www.RibosomalProteins.com).

While only a few r-proteins are required for nucleolar structure maintenance, most of them are essential to pre-rRNA processing (Fig. 4). The processing steps in which r-proteins are involved are primarily those which lead to synthesis of the rRNAs constituting the subunit to which they belong. The r-proteins whose depletion has the strongest impact on nucleolar structure are required for late processing reactions in the pathway of LSU synthesis (Fig. 2a,b).

Pre-rRNA processing is an excellent proxy of ribosome assembly. Hence, by establishing the precise involvement of each r-protein in processing, we incidentally extend the conclusion^23^,^28^,^30^,^31^,^48^ that the sequence of r-protein incorporation into maturing ribosomal subunits, and thus of ribosomal landmark formation, has been extremely well conserved throughout evolution from bacteria, to yeast and man (Supplementary Fig. 10). Importantly, we reveal this to be the case for human large ribosomal subunit assembly. This is quite remarkable, considering the tremendous differences in cell organization, in gene expression strategies and the increased complexity in ribosomal assembly machineries between prokaryotes and eukaryotes.

On mature 60S, the r-proteins whose depletion has the strongest impact on nucleolar structure form specific landmarks: the CP (uL5 and uL18), the L1-stalk and a region directly below the L1-stalk (Fig. 2). These are late-assembling subunit structures. Furthermore, nucleolar structure is disrupted when uL5 or uL18 incorporation, and hence formation of the CP, is prevented by depletion of specific CP assembly factors (Fig. 5). We speculate that the importance of these r-proteins in maintaining the integrity of nucleolar structure reflects the emergence, during evolution, of checkpoints important to cell homeostasis, ensuring that the late steps of LSU assembly, and particularly CP formation, occur properly.

Why should CP formation be monitored? First, in the mature ribosome, the CP is involved in intersubunit interactions beneficial to translation^49^,^50^. Furthermore, CP formation might be tightly coupled to maturation of essential ribosomal landmarks on the LSU. This is plausible, given what is known about CP formation. The 5S ribonucleoprotein is incorporated into maturing 60S subunits as a pre-assembled block^41^, a step aided by the conserved assembly factors Rpf2(yeast)/BXDC1(human) and Rrs1/RRS1 (refs 26, 41, 51; see above). In precursor 60S, however, the 5S ribonucleoprotein does not adopt its final conformation until it undergoes a 180° rotation^51^,^52^. This rotation seems to act as a power stroke promoting a cascade of subunit maturation events and the long-range transmission of mechano-chemical remodelling energy throughout the maturing 60S precursors^52^,^53^. Structures whose formation may be strictly linked to that of the CP include the conserved A-site finger helix 38, which is part of an intersubunit bridge that monitors the A-site transfer RNA throughout the decoding process^54^, the peptidyl transferase centre itself, where amino acids are joined together, and possibly the phospho-stalk^52^,^53^.

It is now well established that several r-proteins are essential to regulating the p53 level (summarized in Supplementary Data 4)^9^,^43^. In principle, depletion of any r-protein is expected to trigger a ribotoxic stress-response leading to accumulation of unassembled ribosomal components, including uL5, uL18 and the 5S rRNA, and to sequestration of Hdm2. Therefore, it was expected that most r-proteins would be involved, in one way or another, in regulating the p53 level. In fact, setting a fivefold increase as significance threshold, we reveal that depletion of any one among two-thirds of the human r-proteins has no significant impact on p53 accumulation (Fig. 6). Nonetheless, we show that 24 r-proteins out of 80 are very important for p53 homeostasis, their depletion giving rise to a 5- to 10-fold increase in the p53 level. In all of these cases, the increase in p53 accumulation requires the presence of uL5 and uL18 (Supplementary Fig. 9). This implies activation of the anti-tumor nucleolar surveillance regulatory loop described above. The r-proteins whose depletion has the strongest impact on p53 homeostasis correspond to late-assembling structures on the subunits (Fig. 2b,c). Identification of the r-proteins whose depletion affects p53 accumulation provides essential insights into the aetiology of ribosomopathies, which are cancer predisposition syndromes caused by mutations in r-proteins or by ribosome assembly defects^7^.

Up to now, it has been unclear whether the activation of nucleolar surveillance, leading to p53 stabilization, systematically involves disruption of nucleolar structure or simply inhibition of nucleolar function. Induction of p53 in response to ribosome biogenesis inhibition has indeed been attributed to nucleolar disruption^55^, but studies have also shown that a rise in the cellular level of p53 can occur after r-protein depletion, independently of gross nucleolar disruption^44^,^56^. This is notably the case after uL30 (formerly: RpL7) depletion^44^. We have confirmed this latter observation, showing that it applies, in fact, to a large group of 21 r-proteins (Supplementary Data 5).

The nucleolus is a long-known cancer biomarker^17^ and a recently demonstrated therapeutic target^19^. It is not widely used by pathologists, however, for lack of reliable clinical assays. The image-processing algorithm and iNo developed here are robust and versatile tools for characterizing nucleolar morphological alterations both qualitatively and quantitatively. We have used them consistently in multiple cell lines, in time course analyses, and with either the dense fibrillar component or the granular component of the nucleolus (Fig. 3, Supplementary Figs 3 and 4). We believe they hold great diagnostic and prognostic potential in cancer biology and research on ribosomopathies, several of which involve marked disruption of nucleolar integrity due to r-protein loss or mutation.

The complete data set and additional information are accessible in a fully searchable information-rich database at www.RibosomalProteins.com.

## Methods

### Nucleolar screens

The nucleolar screens were performed on an automated high-throughput platform. For each r-protein, three different siRNAs were used, and for each siRNA, 2,000 cells imaged. For consistency, the entire screen was duplicated. The efficiency of siRNA-mediated depletion was assessed in a random shotgun RT–qPCR assay. A calibration set consisting of four control proteins whose depletion, we established, affects strongly nucleolar structure was used (Supplementary Fig. 1).

### Cell lines

The cell lines used in this study are listed in Supplementary Data 1. All cell lines were cultured at 37 °C under 5% CO_2_. Culture media were supplemented with 10% foetal bovine serum (Sigma) and 1% penicillin–streptomycin (Pen-Strep, Gibco). For consistency, the experiments were performed on cells grown for 10–15 passages. The nucleolar screens were conducted in cervical cancer (HeLa) cells stably expressing FBL in fusion with GFP (FIB364). All cell lines were purchased from the ATCC repository and regularly tested for contamination with the LookOut mycoplasma PCR detection kit (Sigma-Aldrich, MP0035)

### siRNA depletion

The FIB364 cell line was transfected with either of three distinct siRNAs targeting each r-protein according to the protocol described below (Supplementary Data 2 for siRNA sequences). The entire screening procedure was duplicated. Depletions were performed in 96-well plates (Porvair Sciences). A transfection reagent mix (0.125 μl of Interferin and 20 μl of Optimem) was added to each plate well and left to set for 10 min at room temperature (RT). siRNA (10 μl of 100 nM stock) were added to this mix and left to set for another 30 min at RT. Cells (70 μl of 100,000 cells ml^−1^) were added to each well and the plates were incubated for 3 days. For each individual plate, a set of 7 wells was used for negative and positive controls. Our calibration set consists of mock-treated cell (cells with the transfection reagent mix only) and cells treated with a non-targeting siRNA scramble (SCR), or with siRNA specific to GFP, FBL, nucleophosmin, nucleolin or TIF1A. Cells were fixed in 2% formaldehyde, washed in PBS, incubated 10 min in the presence of DAPI (1:20,000 of 5 mg ml^−1^ in PBS, Sigma), washed again and stored in PBS before imaging. The depletions of the central protuberance assembly factors RRS1 and BXDC1 were performed according to the same protocol.

### Imaging

Imaging was performed on a Zeiss Axio Observer.Z1 microscope with a motorized stage, driven by MetaMorph (MDS Analytical Technologies, Canada). Images were captured in widefield mode with a × 20 objective (Plan NeoFluar, Zeiss), a LED illumination (CoolLed pE-2) and a CoolSnap HQ2 camera. Sixteen independent fields of view were captured automatically for each well. The correct focal plane was maintained by using the built-in autofocus module of MetaMorph. High-resolution images were captured in confocal mode using a Yokogawa spindisk head and the HQ2 camera with a laser from Roper (405 nm 100 mW Vortran, 491 nm 50 mW Cobolt Calypso and 561 nm 50 mW Cobolt Jive) and a × 40 objective (Plan NeoFluar, Zeiss).

### PES1 detection by indirect immunofluorescence

After 3 days siRNA-mediated depletion, cells were fixed in 2% formaldehyde, washed in PBS and blocked in PBS supplemented with 5% BSA and 0.3% Triton X-100 during 1 hour at RT. Anti-PES1 antibody (anti-rat, 1:1,000; courtesy from E. Kremmer) was diluted in PBS supplemented with 1% BSA, 0.3% Triton X-100 and incubated with the cells overnight at 4 °C. Cells were washed in PBS and incubated with a secondary Alexa Fluor 594 anti-rat antibody (1:1,000; Invitrogen) in PBS, 1% BSA, 0.3% Triton X-100 during 1 h at RT. Cells were finally washed in PBS, treated with DAPI and imaged with the Zeiss microscope as described above.

### Image processing and iNo index

The supplemental section of our manuscript presents our methodology for distinguishing populations of normal and altered nucleoli, based on statistical morphometric information (Supplementary Tables 1 and 2, and Supplementary Figs 14–22). Shape and textural features were first derived to characterize nucleolar morphology in individual cell nuclei, so as to distinguish normal from altered nucleoli morphology in FIB-GFP images. Each feature was systematically defined as a parametric function, so that its parameters could be optimized over the entire database to maximize Fisher's criterion computed between the distributions of the features observed in r-protein-depleted cells and SCR-treated control cells. Given these features, we then performed a quantitative analysis of differences between their statistical distributions in a population of r-protein-depleted cells, compared with their distributions in a reference population. For this, we introduced a so-called discrepancy vector, each component of this vector being associated with a specific feature, and measured the distance between the distribution observed for a population of cells depleted of a given r-protein and that observed for a reference population of cells (SCR-treated cells). We then defined the iNo, as the L1-norm of the discrepancy vector. This index reflects the degree of severity of nucleolar disruption, that is, it ranks the r-proteins according to their impact on nucleolar structure. Additionally, PCA of the discrepancy vectors was used to extract and visualize the major trends affecting the morphology of the nucleolus on gene product depletion. PCA assumes linear embedding for dimensionality reduction and allows unsupervised clustering of the nucleolar disruption phenotypes. The computer code is described in the Supplementary Information section and available on request.

### Pre-rRNA processing analysis

For the pre-rRNA analysis, we used a colon carcinoma cell line (HCT116) expressing normally p53 (ref. 39). Northern blot analyses were performed essentially as described in ref. 38 and www.RibosomeSynthesis.com. Briefly, HCT116 cells were transfected with one siRNA specific to transcripts encoding each r-protein in 6-well plates and incubated for 2 days before total RNA extraction and northern blot analysis. The probes used are described in Supplementary Data 3. Two distinct siRNAs were used in two independent experiments. The ‘R' software was used to generate and cluster the heatmaps. These heatmaps are a visual representation of the logarithm of the ratio of the pre-rRNA level in the knockdown condition respective to its level in the non-targeting (Scramble, SCR) control. The calibration set used in the pre-rRNA processing analysis consists of mock-treated cells, and cells treated with a non-targeting siRNA (Scramble, SCR), or with siRNAs specific to UTP18 or NOL9 (Supplementary Data 2; ref. 38 and www.RibosomeSynthesis.com). In our clustering analysis, we did not average the processing data obtained with the two different siRNAs used in this work for each r-protein, but rather, we considered them as individual experiments. In most cases, the two independent processing data sets obtained for any particular r-protein are highly clustered, demonstrating the robustness of our screens. In a few cases (denoted with a star in Fig. 4, and observed only for two SSU and six LSU r-proteins), the heatmaps do not belong to the same class, reflecting the inherent variation in depletion efficiency from one individual siRNA to another. Note that all RNA species detected were used to cluster the heatmaps shown in Fig. 4c,d but only those directly relevant to synthesis of the small (in Fig. 4c), or large (in Fig. 4d) subunit are shown for simplicity. The clusters with all the RNA species are shown in Supplementary Figs 5 and 6, and on www.RibosomalProteins.com. The 28S/18S rRNA ratios were calculated from Agilent bioanalyzer electropherograms according to the manufacturer's instructions. Examples of uncropped northern blots are show in Supplementary Fig. 12.

### p53 steady-state level analysis

For p53 steady-state analysis, we used a colon carcinoma cell line (HCT116) expressing p53 (ref. 39). For quantitative western-blot analysis, HCT116 cells were depleted three times independently with one siRNA specific to transcripts encoding each r-protein. The transfection protocol used was similar to the one described above in the rRNA processing analysis section. For total protein extractions, cells from 6-well plates were first detached with 300 μl of trypsin-EDTA (ATCC) and pelleted at 100 g for 10 min at RT. Cells were washed in 1 ml of cold PBS and pelleted again at 100 g for 10 min at RT. Cells were then lysed in 30 μl of lysis buffer (Tris-HCl pH 8.0, 20 mM; NP40, 0.5%; NaCl, 150 mM; EDTA, 1 mM, protease inhibitor-Roche) during 15 min on ice. Lysed cells were then centrifuged at 20,000*g* for 10 min at 4 °C and supernatants were recovered from the pellet of cellular debris. As controls, we used the non-targeting scramble siRNA and an antisense oligonucleotide targeting the U8 snoRNA (IDT; Supplementary Data 2). Forty microgram of total protein were separated on a 4–12% polyacrylamide gel (Novex, Life Technologies, Bolt Bis-Tris Plus) and transferred on low-fluorescence polyvinylidene fluoride (PVDF) membrane (Immobilon-FL, Millipore) according to the manufacturer protocol. The membranes were blocked in Odyssey blocking buffer (Li-Cor) for 1 h at RT. Primary antibodies (1:4,000 anti-β-actin, Santa Cruz, SC69879; and 1:1,000 anti-p53, Bethyl Laboratories, A300-247A) were added to the Odyssey blocking buffer supplemented with 0.2% Tween-20 (Sigma) and membranes were incubated overnight at 4 °C with agitation. Membranes were washed three times in tris-buffered saline (TBS) supplemented with 0.1% Tween-20 (TBS-T). Secondary antibodies carrying fluorescent dyes (1:2,000 DyLight 550 anti-mouse, Thermo Scientific, 84540; and 1:2,000 IRDye 680 anti-rabbit, Westburg, 926-68071) were added to Odyssey blocking buffer supplemented with 0.1% SDS and 0.2% Tween-20 and membranes were incubated 1 h at RT with agitation. Membranes were washed three times in TBS-T before imaging of the fluorescent signals with the Chemidoc (Biorad). Cellular p53 steady-state level was assessed by calculating a ratio between the red fluorescent signal (corresponding to p53) and the green fluorescent signal (corresponding to β-actin). For each experiment, two independent lanes corresponding to HCT116 cells treated with the SCR siRNA were loaded on the gel, and the results from these two lanes were averaged to determine the level of p53 in this control condition. All data were then harmonized to this averaged value to determine the variation in the p53 steady-state level under this reference condition. Examples of uncropped western blots are shown in Supplementary Fig. 13. In Supplementary Fig. 9, the western blots were performed according to the same protocol, except that the gels were transferred onto PVDF (Amersham Hybond-P, RPN303F), and revealed with an HRP-conjugated secondary antibody (Santa Cruz) and the Supersignal WestPico chemiluminescent ECL substrate (Thermo Scientific). The anti-p21 antibody was purchase from Cell signalling (2947S).

For 48 r-proteins whose depletion did not affect the p53 level, the residual mRNA level was established by RT–qPCR (Supplementary Fig. 8). Reverse transcription was performed with the qScript cDNA supermix (Quanta Biosciences). qPCR was performed on a StepOne Plus Real-Time PCR machine (ThermoFisher Scientific) with specific primer pairs (Supplementary Table 3) and the perfecta SYBR green supermix (Quanta Biosciences). Each reaction was performed in triplicate. The residual level of mRNA was normalized to that of GAPDH and expressed with respect to that observed in cells treated with a non-targeting siRNA control (SCR).

## Additional information

**How to cite this article:** Nicolas, E. *et al*. Involvement of human ribosomal proteins in nucleolar structure and p53-dependent nucleolar stress. *Nat. Commun.* 7:11390 doi: 10.1038/ncomms11390 (2016).

## Supplementary Material

Supplementary Information Supplementary Figures 1-23, Supplementary Tables 6-7, Supplementary Note 1, Supplementary Methods and Supplementary References

Supplementary Data 1 Cell lines

Supplementary Data 2 siRNA

Supplementary Data 3 Oligonucleotides used in NB and RTqPCR

Supplementary Data 4 Comparison with literature

Supplementary Data 5 p53 and nucleolar integrity
