# Supplementary material for: Involvement of human ribosomal proteins in nucleolar structure and p53-dependent nucleolar stress
Source: Nat Commun. 2016 Jun 6;7:11390. doi: 10.1038/ncomms11390 (PMC4897761; doi:10.1038/ncomms11390)
Supplement: Supplementary Information — Supplementary Figures 1-23, Supplementary Tables 6-7, Supplementary Note 1, Supplementary Methods and Supplementary References [file ncomms11390-s1.pdf]

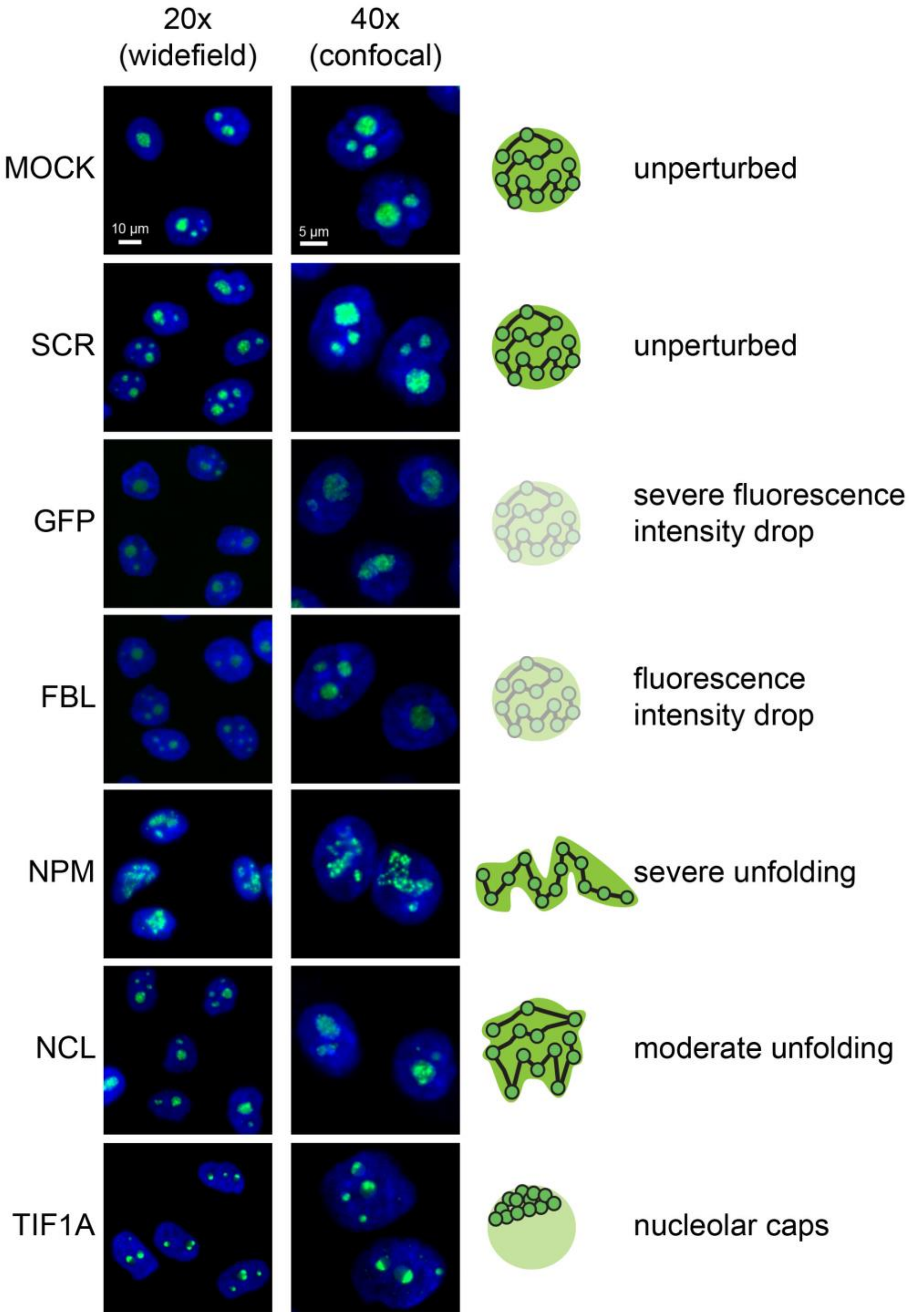

Supplementary Fig. 1: The nucleolar calibration set
The calibration set used consisted of four control proteins whose depletion, we established, strongly disrupts nucleolar structure. These control proteins are: the RNA polymerase I (Pol I) transcription factor TIF1A, nucleolin (NCL), and nucleophosmin (NPM). As further standardization controls, we used mock-treated (MOCK) cells, cells treated with an siRNA targeting FBL or GFP, and cells treated with a non-targeting siRNA (SCR). Blue signal, DNA stain (DAPI); green signal, GFP. Left column, images captured at 20x magnification in widefield mode. Right column, images captured at 40x magnification in confocal mode. To the right of these images, schematics depicting the effect of siRNA-mediated depletion on nucleolar structure and signal intensity.

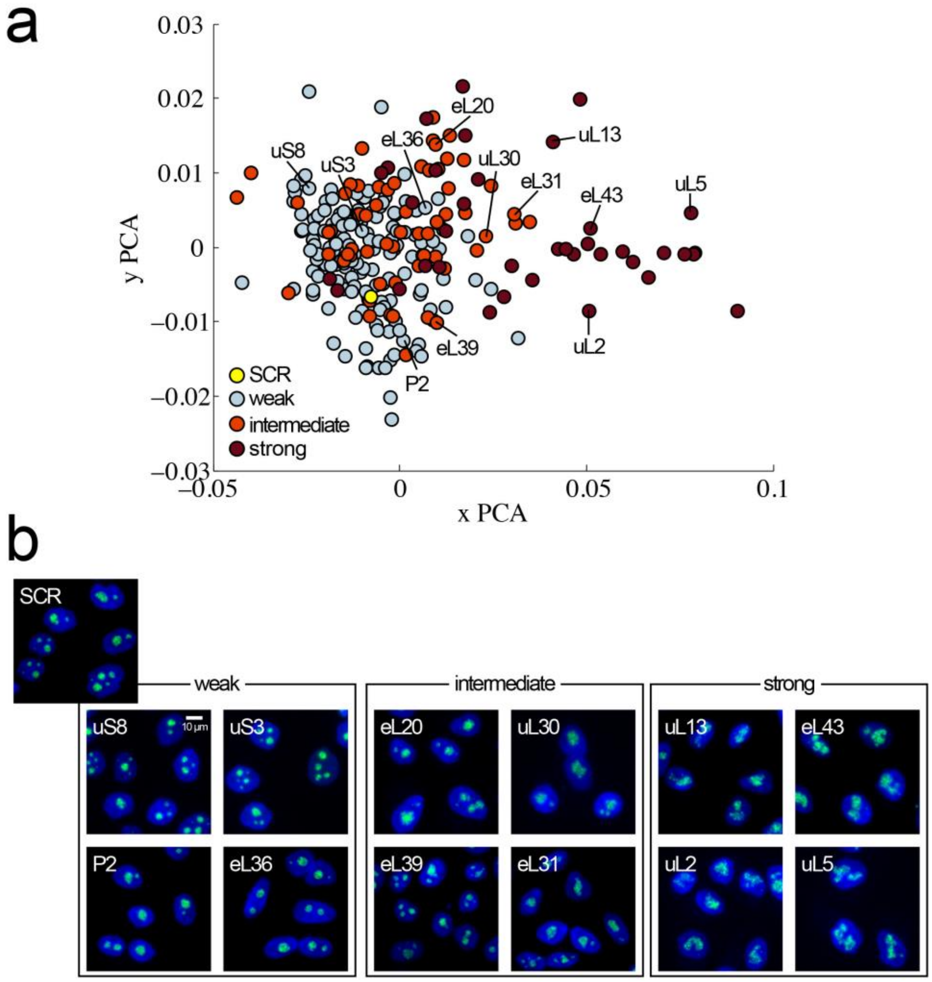

Supplementary Fig. 2: Benchmarking the automated unsupervised classification of nucleolar disruption phenotypes

To benchmark our novel classification algorithm, we compared the automated classification described in the text with a manual one. The manual classification was based on the fine visual inspection of representative images obtained after depletion with each siRNA used and on the assignment of nucleolar disruption phenotypes to three arbitrarily defined classes corresponding to weak, intermediate, and strong disruption. Superimposition of the automatically and manually obtained classifications made us highly confident that assignment to phenotypic classes on the basis of our automated procedure is robust.

(**a**) PCA (see Fig. 1b). Each dot represents a population of cells treated with one siRNA specific to one r-protein. Dots are color-coded according to a manual classification based on the fine visual inspection of the microscopic images. The non-targeting control (Scramble, SCR) is in yellow. Strong contributors to nucleolar structure maintenance are wine-colored; intermediate contributors are in red; weak contributors are in pale blue.

(**b**) Representative images illustrating the manual classification of r-proteins as weak, intermediate, and strong contributors to nucleolar structure maintenance. Blue signal, DNA stain; green signal, fibrillarin.

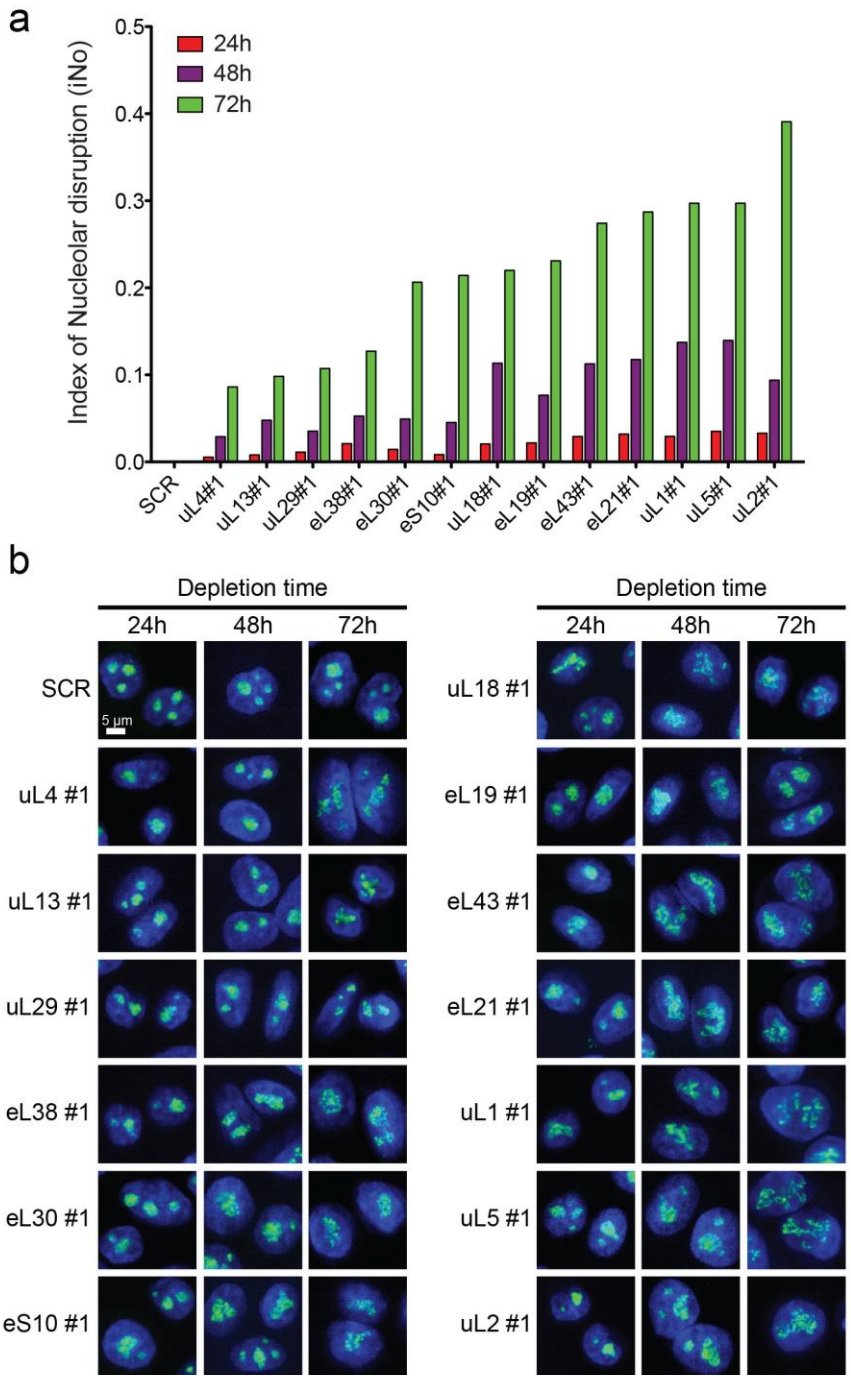

Supplementary Fig. 3: Kinetics of nucleolar disruption after siRNA-mediated r-protein depletion

The data show that the computed iNo values reliably reflect phenotype severity, and that nucleolar disruption is best scored in cells having undergone two successive rounds of nucleolar breakdown/nucleolar genesis, corresponding to two cell divisions (~72h).

(**a**) Values of the nucleolar disruption index (iNo) obtained after siRNA-mediated depletion of the indicated r-protein for 24 h, 48 h, and 72 h.

(**b**) Representative images for thirteen r-proteins tested at each time point. Blue signal, DNA stain; green signal, fibrillarin. #1 refers to the siRNA used (see Methods).

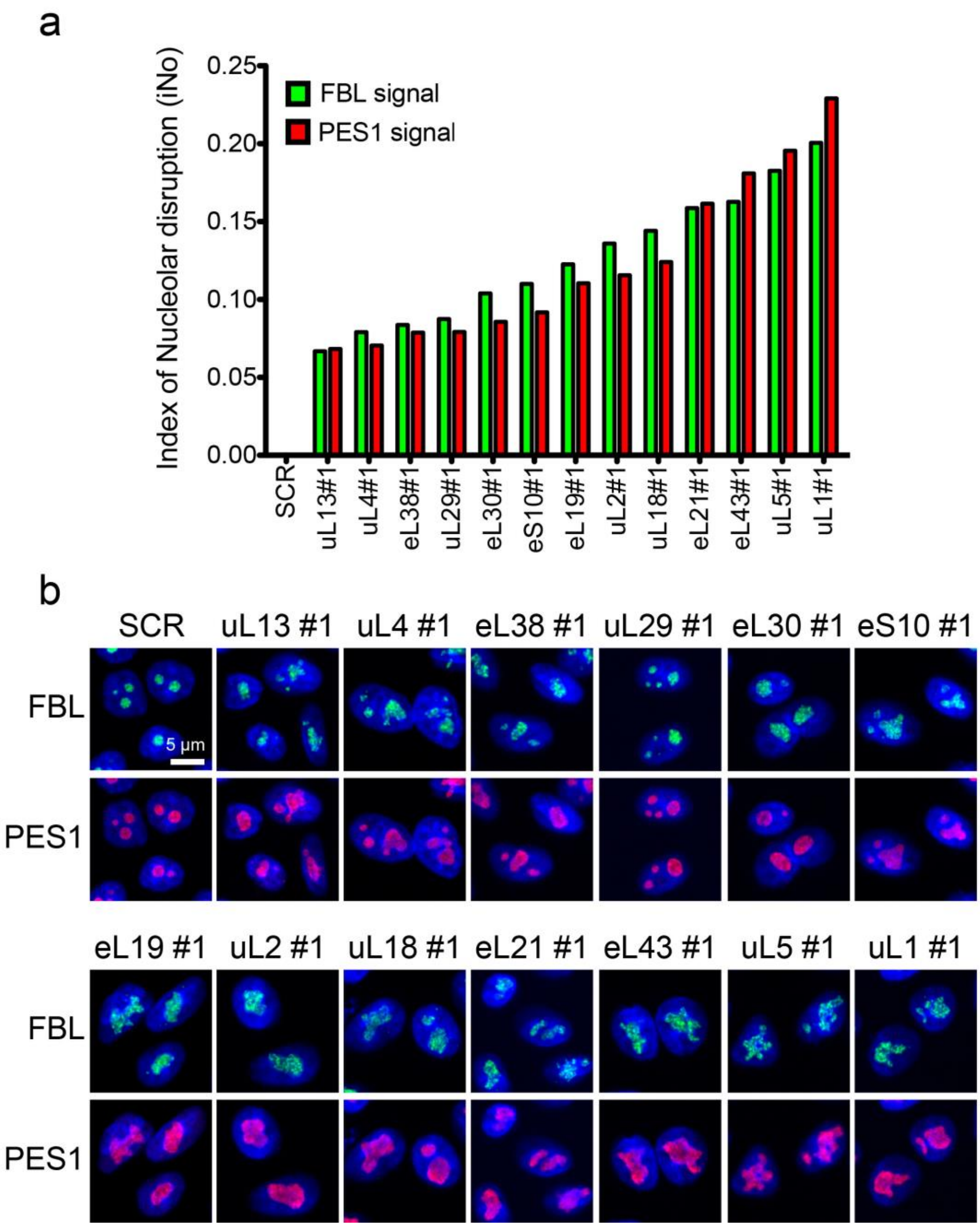

Supplementary Fig. 4: Quantitative monitoring of nucleolar morphology based on detection of the endogenous granular component marker PES1
The data show that the iNo values can be computed equally on the basis of DFC (fibrilarin, FBL) or GC (PES1) antigen detection.
(**a**) Values of the nucleolar disruption index (iNo) obtained after 3 days of siRNA-mediated depletion of the indicated r-protein as calculated on the basis of the fibrillarin signal (in green) or the PES1 signal (in red).
(**b**) Representative images for the thirteen r-proteins tested. In each case the siRNA n°1 (#1) was used (see Methods). Blue signal, DNA stain; green signal, fibrillarin; red signal, PES1.

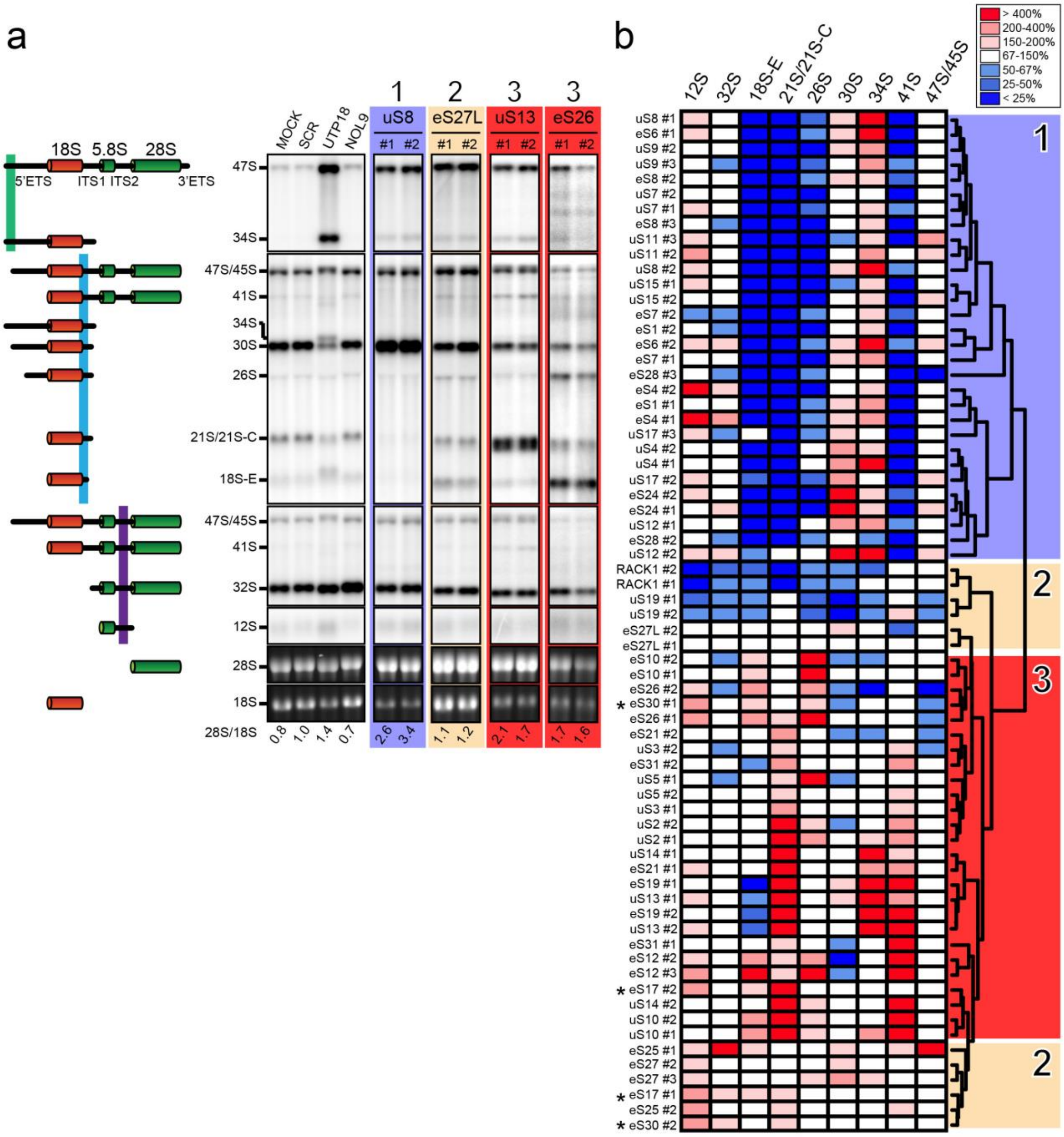

Supplementary Fig. 5: Involvement of small subunit r-proteins in pre-rRNA processing

(**a**) Representative examples of northern blots for each of the three classes of SSU r-proteins defined in this work (for a full dataset see www.RibosomalProteins.com and Supplementary Fig. 11). A calibration set consisting of mock-treated cells and cells treated with a non-targeting siRNA (SCR) or a siRNA targeting UTP18 or NOL9 was used systematically (see ref.[1]). Schematics of the RNA intermediates detected are shown on the left. Ratios of 28S to 18S mature rRNA were calculated from bioanalyzer electropherograms. (**b**) Expanded version of Fig. 4c, showing all RNA intermediates detected and quantified.

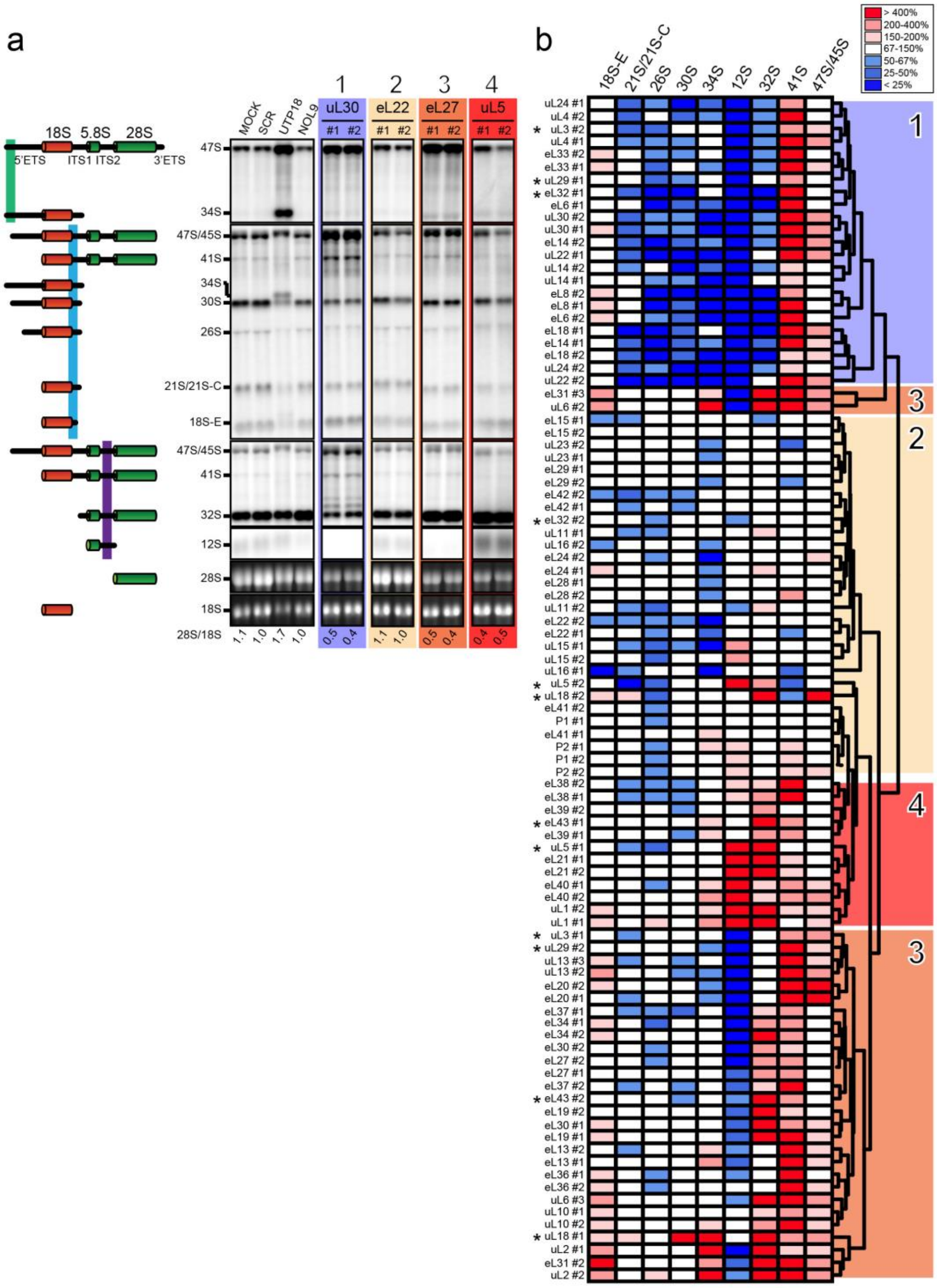

Supplementary Fig. 6: Involvement of large subunit r-proteins in pre-rRNA processing

(**a**) Representative examples of northern blots for each of the four classes of LSU r-proteins defined in this work (for a full dataset see www.RibosomalProteins.com and Supplementary Fig. 11). A calibration set consisting of mock-treated cells and cells treated with a non-targeting siRNA (SCR) or a siRNA targeting UTP18 or NOL9 was used systematically (see ref.[1]). Schematics of the RNA intermediates detected are shown on the left. 28S/18S mature rRNA ratios were calculated from bioanalyzer electropherograms.

(**b**) Expanded version of Fig. 4d, showing all RNA intermediates detected and quantified.

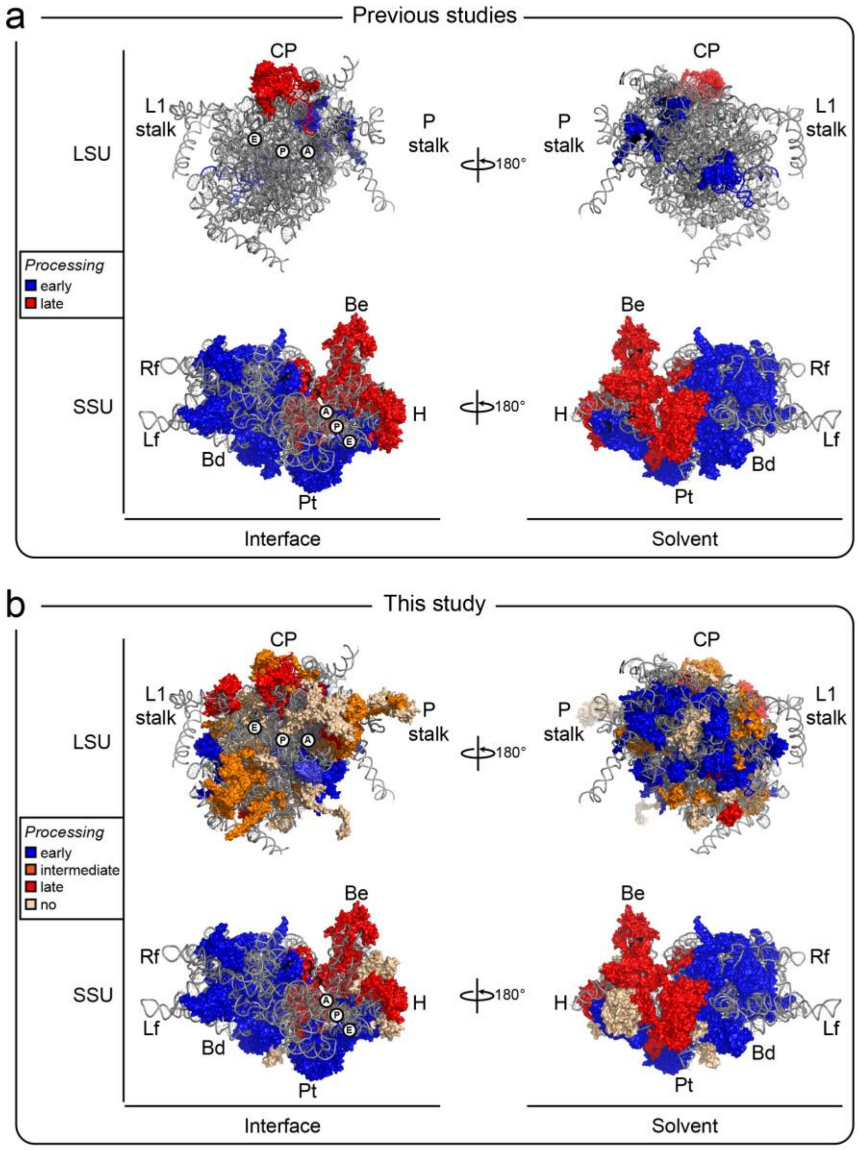

Supplementary Fig. 7: Comparison of our classification of r-proteins according to their involvement in pre-rRNA processing with previous studies

The figure shows that our work either confirms (small subunit r-proteins) or considerably complements (large subunit r-proteins) the literature.

3-D models of human ribosomal subunits based on PDB entries 3J3D, 3J3A, 3J3F, and 3J3B. Left, subunit interface views; right, solvent-exposed views. The aminoacyl (A), peptidyl (P), and exit (E) tRNA sites are indicated. Morphological features of the subunits are highlighted. On the LSU: the L1-stalk, central protuberance (CP), and phospho-stalk (P-stalk). On the SSU, the beak (Be), head (H), platform (Pt), body (Bd), left foot (Lf), and right foot (Rf).

(**a**) Previous studies: conducted on cervix cancer cells where p53 expression is disrupted by HPV integration (HeLa cells). The SSU r-proteins were tested in ref. [2]; six out of the forty-seven LSU r-proteins were tested in ref. [3].

(**b**) This work: conducted on colon carcinoma cells expressing p53 normally (HCT116 p53$^{+/+}$)(based on Fig. 4 and Supplementary Figs. S5,S6,S11). All r-proteins were tested.

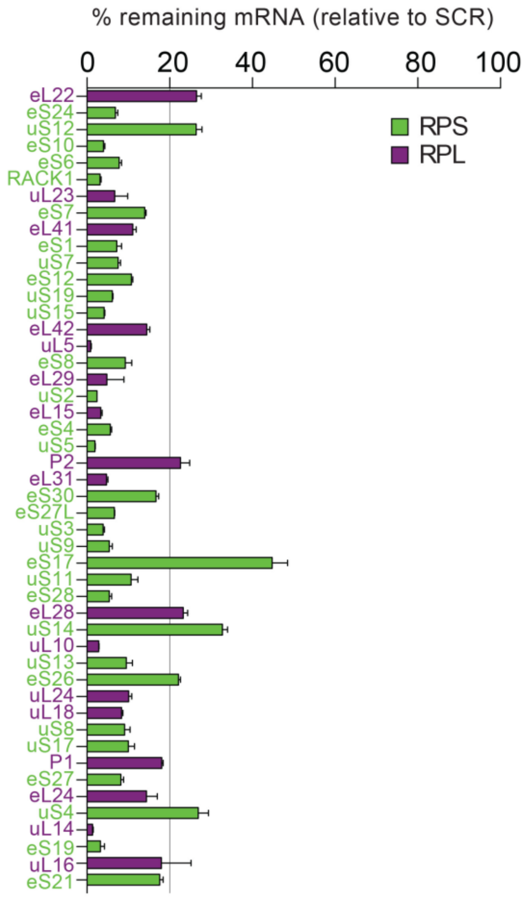

Supplementary Fig. 8: Efficiency of r-protein depletion established at the mRNA level by RTqPCR

For forty-eight r-proteins whose depletion did not significantly affect p53 accumulation (see Fig. 6b), the residual level of mRNA was established by RTqPCR and found to be below 20% for forty of them, and to range between 20% and 45% for the remaining eight. Total RNA was extracted from HCT116 cells treated for 2 days with an siRNA specific to transcripts encoding the indicated r-proteins. Residual levels of mRNA were established by RTqPCR and normalized to those observed in cells treated with a non-targeting siRNA control (SCR). Each experiment was performed in triplicate.

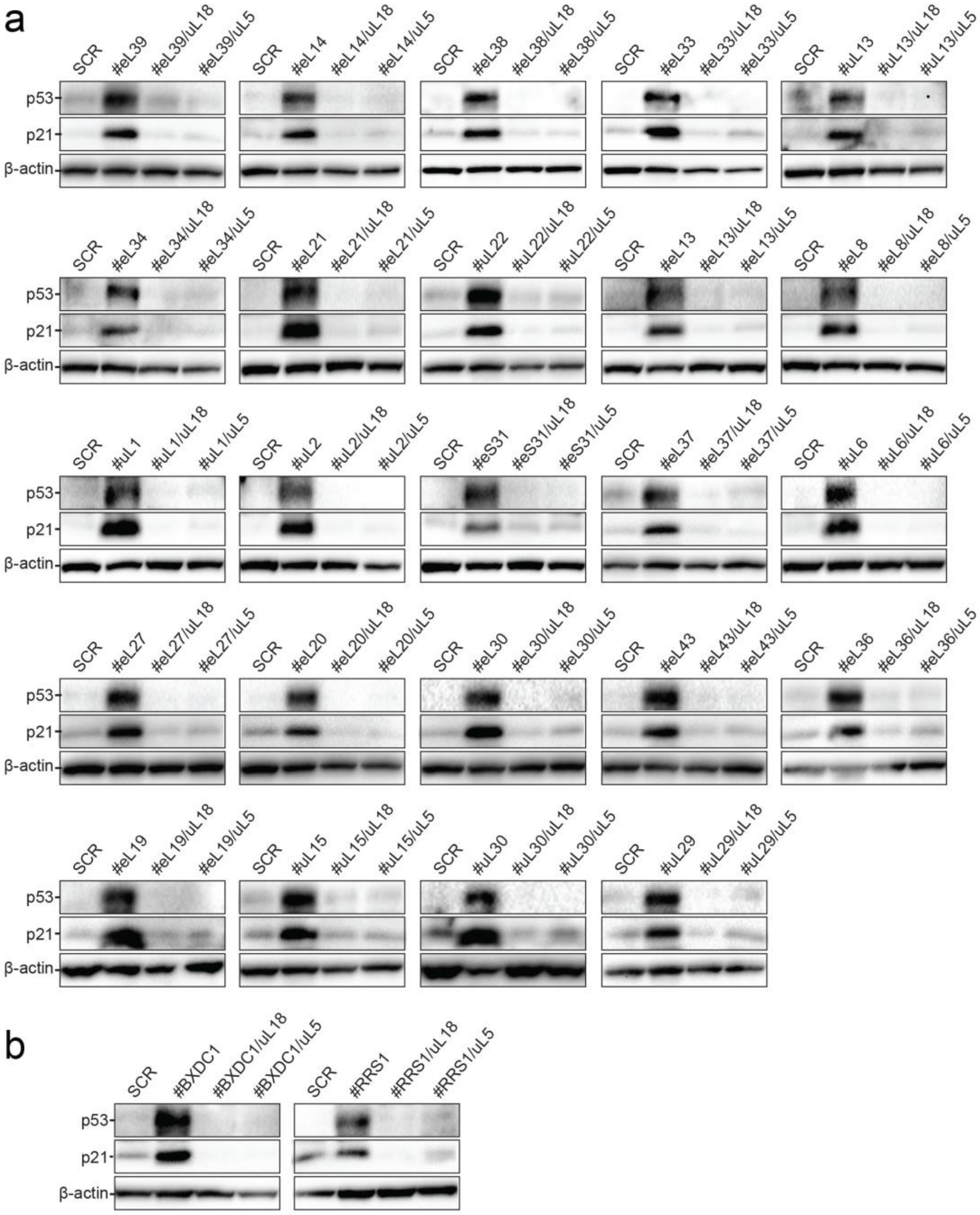

Supplementary Fig. 9: Depletion of twenty-four r-proteins out of eighty leads to a significant five-fold-increased level of p53, and this increase requires the presence of uL5 and uL18

HCT116 p53$^{+/+}$ cells were depleted for 2 days with an siRNA specific to transcripts encoding the indicated protein. Each protein was depleted by itself, or in combination with uL18 or uL5 depletion. As control, cells were treated with a non-targeting siRNA (SCR). Total protein was extracted and processed by western blotting with antibodies targeting p53, p21 (a transcriptional target of p53), and, as loading control, β-actin. Bands were reveled by luminescence.

(**a**) Effect of individually depleting each of the 24 r-proteins

(**b**) Effect of depleting the central protuberance assembly factor BXDC1 or RRS1.

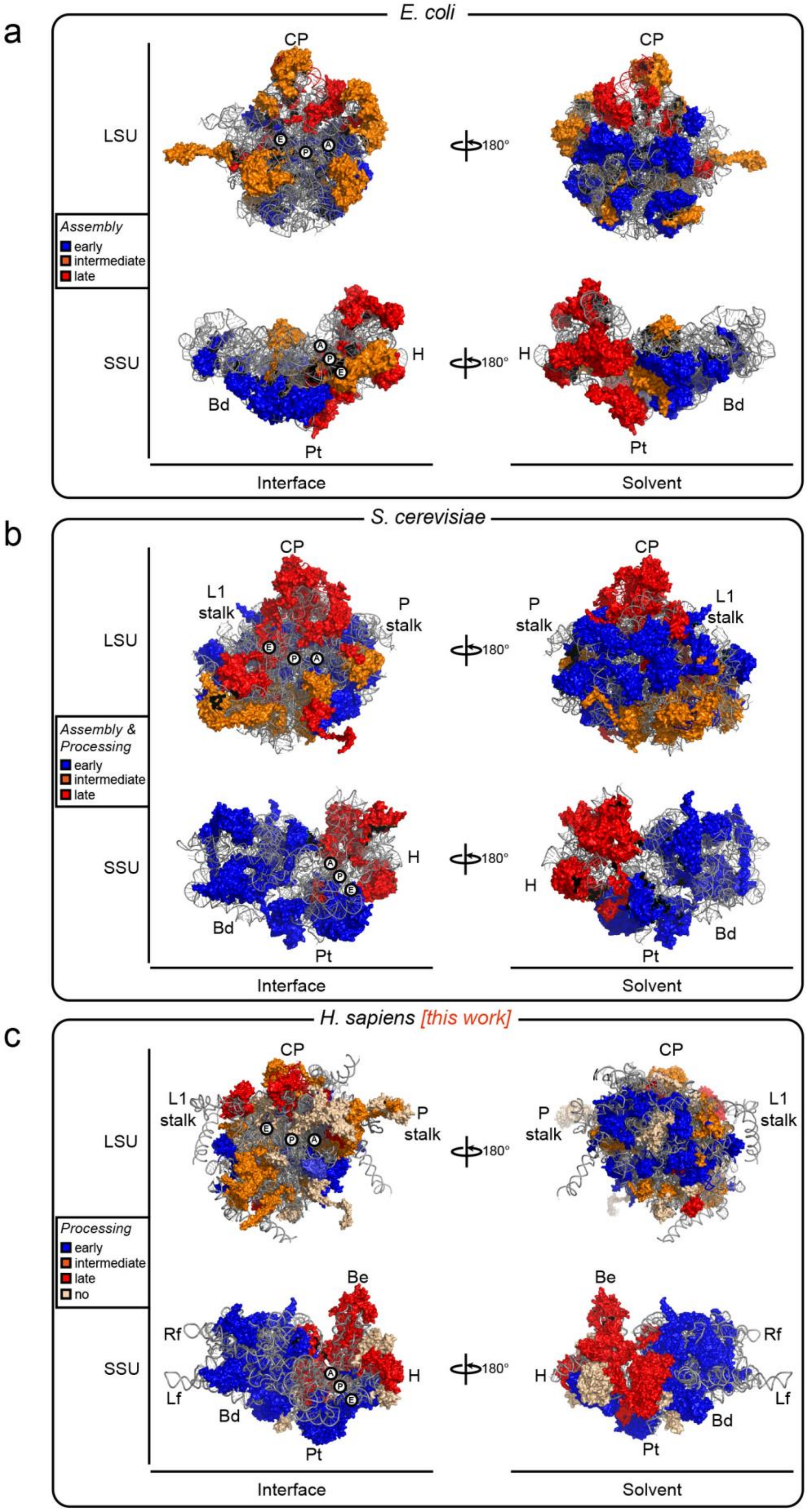
a
E. coli
CP
LSU
180°
Assembly
early
intermediate
late
SSU
H
Bd
Pt
Interface
Solvent
b
S. cerevisiae
L1 stalk
P stalk
Assembly & Processing
c
H. sapiens [this work]
Processing
no
Be
Rf
Lf

Supplementary Fig. 10: The sequence of assembly of r-proteins onto assembling ribosomal subunits has been remarkably conserved throughout evolution

(**a**) 3-D models of bacterial ribosomal subunits based on PDB entries 2AVY and 2AW4. The r-proteins are color-coded according to their early, intermediate, or late order of assembly, as established *in vitro* (refs [4-8]).

(**b**) 3-D models of budding yeast ribosomal subunits based on PDB entries 3U5B, 3U5C, 3U5D, and 3U5E. The r-proteins are labeled on the basis of their being required for specific early, intermediate, or late pre-rRNA processing steps in yeast cells (refs [9, 10]). This largely corresponds to their kinetics of assembly established *in vivo* (ref. [11]).

(**c**) 3-D models of human ribosomal subunits based on PDB entries 3J3D, 3J3A, 3J3F, and 3J3B. The r-proteins are classified with respect to their impact on specific processing reactions in human cells according to this work (based on Fig. 4, Supplementary Figs. S5,S6,S11).

Left, subunit interface views; right, solvent-exposed views. The aminoacyl (A), peptidyl (P), and exit (E) tRNA sites are indicated. Morphological features of the subunits are highlighted. On the LSU: the L1-stalk, central protuberance (CP), and phospho-stalk (P-stalk). On the SSU, the beak (Be), head (H), platform (Pt), body (Bd), left foot (Lf), and right foot (Rf).

eS1 (RpS3a)

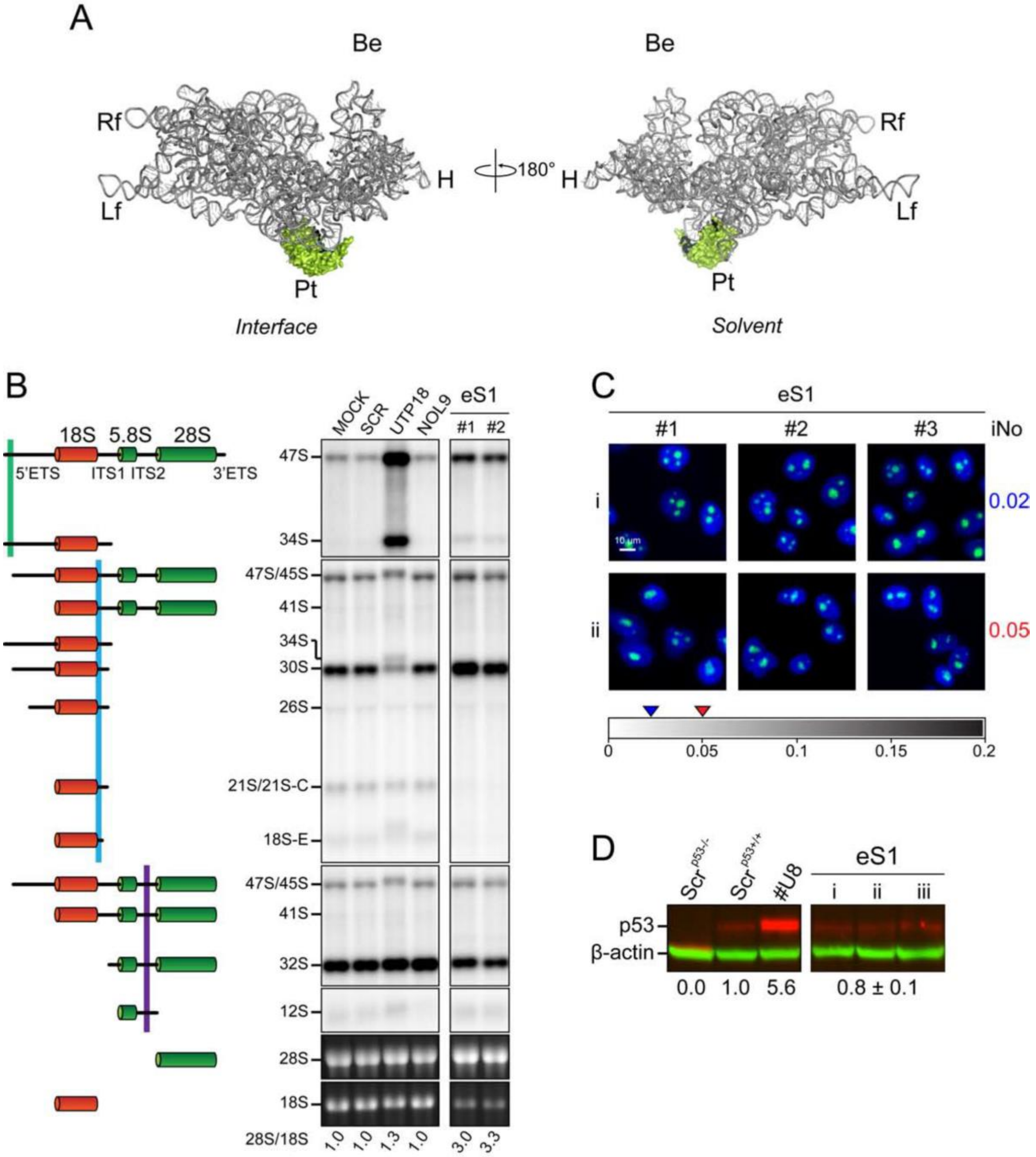
A
Be
Rf
Lf
H
180°
Pt
Interface
Solvent
B
18S 5.8S 28S
5'ETS ITS1 ITS2 3'ETS
MOCK SCR UTP18 NOL9
eS1
#1 #2
47S
34S
47S/45S
41S
34S
30S
26S
21S/21S-C
18S-E
47S/45S
41S
32S
12S
28S
18S
28S/18S
1.0 1.0 1.3 1.0 3.0 3.3
C
eS1
#1 #2 #3
iNo
i
0.02
ii
0.05
10 µm
0 0.05 0.1 0.15 0.2
D
Scr p53-/-
Scr p53+/+
#U8
eS1
i ii iii
p53
β-actin
0.0 1.0 5.6 0.8 ± 0.1

Supplementary Fig. 11: An example of a complete data sheet for one r-protein. The datasheets for all the eighty human r-proteins is available on the companion website at Www.RibosomalProteins.Com

The data sheet shows the position of the r-protein on mature subunit (A), its impacts on pre-rRNA processing and mature rRNA accumulation (B), on nucleolar structure (C), and on the p53 steady-state level (D).

(**A**) 3-D models of human ribosomal subunits based on PDB entries 3J3D and 3J3A, for SSU r-proteins, and 3J3F and 3J3B, for LSU r-proteins. The positions of individual r-proteins on the mature subunits are highlighted. On small subunits, the 18S rRNA is shown in gray; on large subunits, the 5S, 5.8S, and 28S rRNAs are shown in red, blue, and gray, respectively. Left, interface view; right, solvent view. The main ribosomal features are indicated (see Supplementary Fig. 10).

(**B**) Effects of r-protein depletion on pre-rRNA processing and mature rRNA accumulation: northern blots and ethidium-bromide-stained denaturing agarose gels showing all the pre-rRNA intermediates and mature rRNAs detected. The 28S/18S rRNA ratio was calculated from electropherograms. A calibration set (described in Supplementary Figs S5,S6) is included for reference. Schematics representing the RNAs detected are shown to the left. Quantifications are available in Fig 4. and Supplementary Figs S5,S6.

(**C**) Effect of r-protein depletion on nucleolar structure: representative microscopic images (blue signal, DAPI; green signal, fibrillarin) obtained after treatment in duplicate screens (i and ii) performed with three different siRNAs (#1, #2, and #3). iNo values for each screen are indicated to the right and on a scaled bar at the bottom. The iNo value ranges between 0 (unperturbed nucleolus) and 0.2 (severely disrupted structure).

(**D**) Effect of r-protein depletion on the p53 steady-state level: fluorescent quantitative western blotting was performed in triplicate (i, ii, iii). The p53 signal was corrected for loading, using β-actin detection as a reference, and expressed with respect to the signal obtained in cells treated with a non-targeting (Scr) control (lane 2). The p53 signal was expressed as a mean of three independent experiments (see Fig. 6 for details). A calibration set (described in Fig. 6) is included for reference.

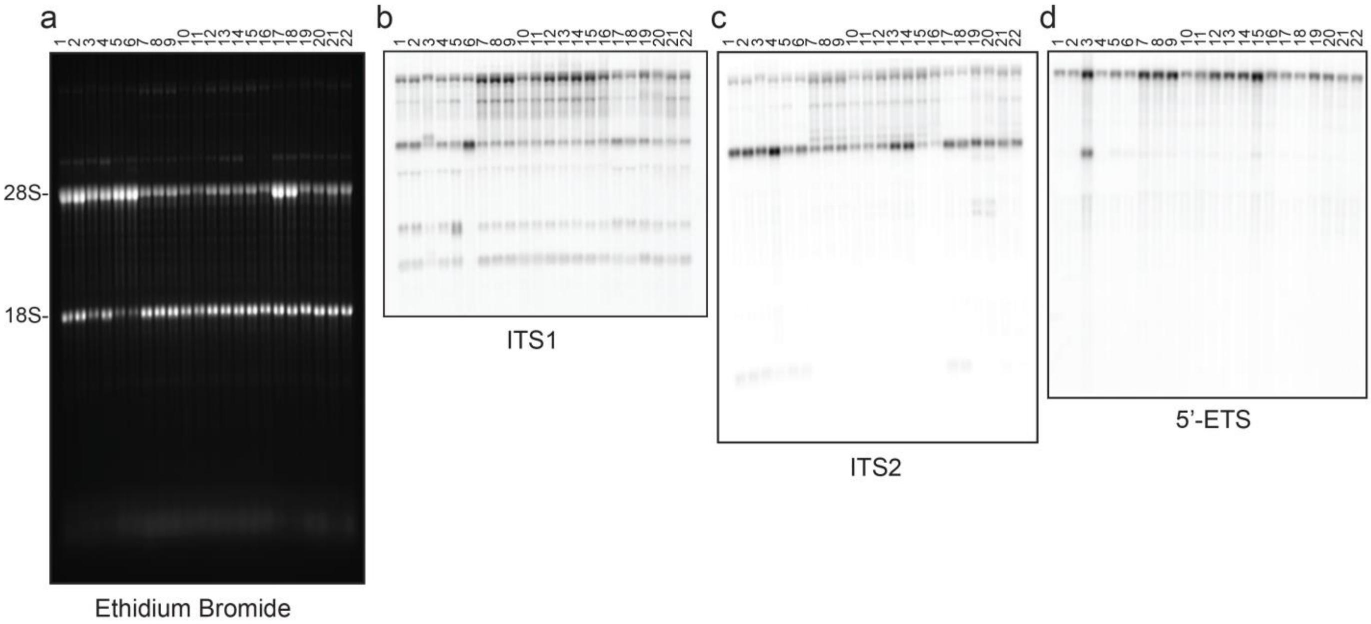

Supplementary Fig. 12: Examples of uncropped Northern blots
An example of a high resolution denaturing agarose gel is shown.
(**a**) Ethidium bromide staining reveals the mature 18S and 28S rRNAs.
(**b**,**c**,**d**) Northern blotting with specific probes reveals the pre-rRNAs. All RNA species were identified by differential hybridization with specific probes, and by reference to a calibration set consisting of two proteins (UTP18 and NOL9) whose depletion leads to well-characterized pre-rRNA processing inhibitions, as described in ref. [1]. The probes used (ITS1 in **b**, ITS2 in **c**, and 5'-ETS in **d**) are described in the methods section.

Lane 1, mock; lane 2, SCR; lane 3, UTP18; lane 4, NOL9; lane 5, uS3; lane 6, uS17; lane 7, uL30#1; lane 8, uL30#2; lane 9, uL13#1; lane 10, uL13#2; lane 11, eL14#1; lane 12, eL14#2; lane 13, ul22#1; lane 14, ul22#2; lane 15, eL18#1; lane 16, eL18#2; lane 17, eL22#1; lane 18, eL22#2; lane 19, uL14#1; lane 20, uL14#2; lane 21, uL24#1; lane 22, uL24#2. #1 and #2 refer to the siRNAs used (see methods section).

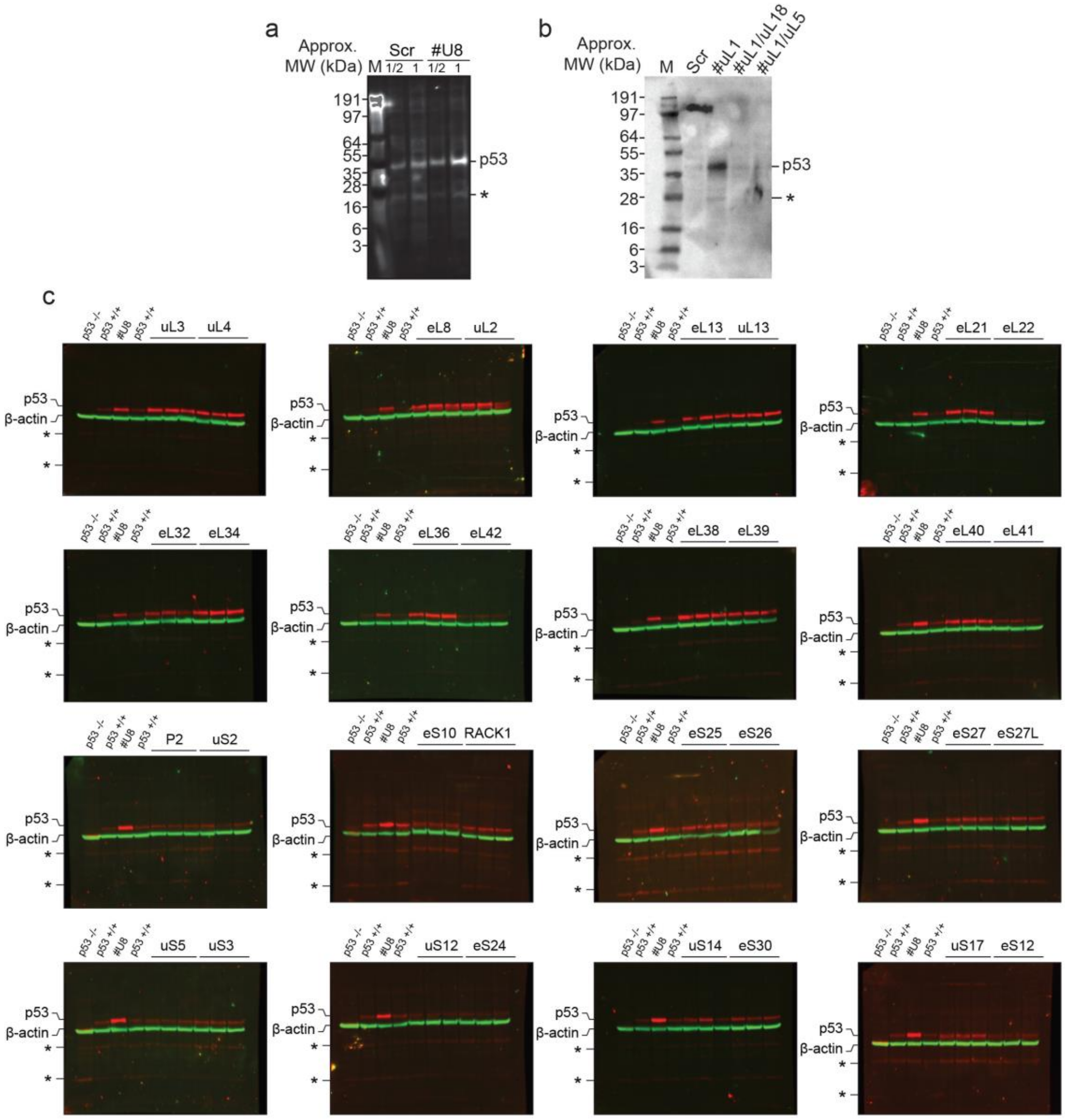

Supplementary Fig. 13: Examples of uncropped Western blots

All western blot hybridizations performed in this work used well-characterized commercially available antibodies (see methods section).

On fluorescent (**a**) and luminescent (**b**) detections, p53 migrated between the 35 kDa and the 55 kDa molecular weight bands, as expected (p53 has a molecular weight of 43.7 kDa). The p53 signal was increased upon nucleolar stress activation (U8 depletion in **a**, and uL1 depletion in **b**, by comparison to the signals observed in cells treated with a non-targeting Scr control). The p53 level was severely reduced upon codepletion of uL1 with uL5 or uL18 (**b**).

On the fluorescent screening gels (representative examples shown in **c**), the β-actin loading control (in green) was detected as a single band immediately below the p53 signal, as expected (β-actin has a molecular weight of 42 kDa). The p53 band (red signal) was consistently detected at low levels in the duplicated HCT116 p53 +/+ cells treated with a non-targeting siRNA (p53 +/+ lanes), this served as a baseline control. The p53 levels increased substantially upon nucleolar stress caused by U8 snoRNA depletion (#U8 lanes), this served as a positive control. The p53 band was never detected in the negative control provided by the HCT116 p53 -/- isogenic cell line (p53 -/-). The stars denote nonspecific bands.

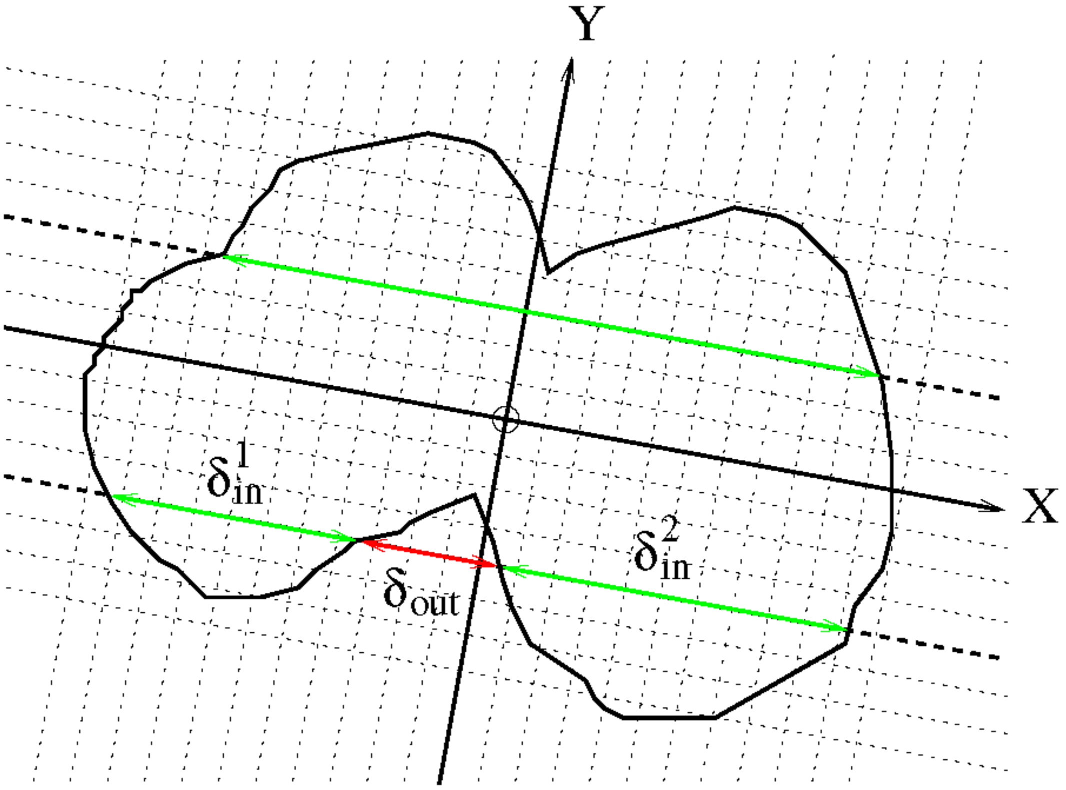

Supplementary Fig. 14: Nuclei aggregates are eliminated on the basis of a shape convexity analysis. Two segmented juxtaposed nuclei are illustrated. In the direction of the principal axis X and Y, if δ_{out}, δ^1_{in}, and δ^2_{in} are above a threshold on one of the lines parallel to the principal axis, the shape is considered to include more than one nucleus.

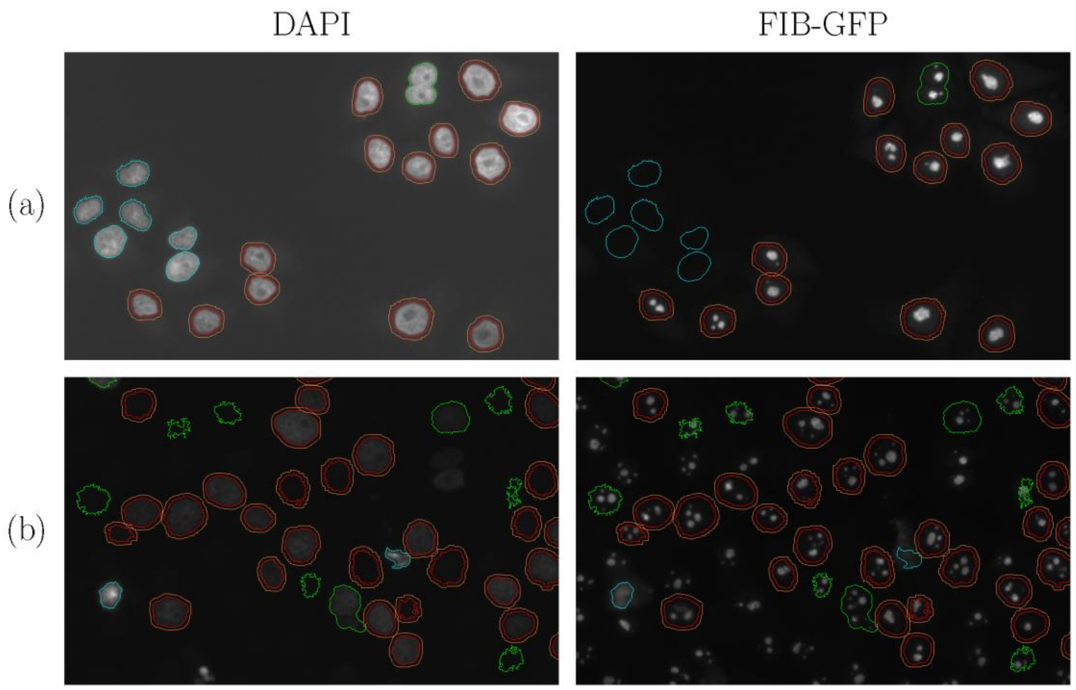

Supplementary Fig. 15: Results of segmentation of nuclei for highly contrasted (**a**) and weakly contrasted (**b**) DAPI images.

Red, green, and cyan contours correspond to the connected components that are detected based on the proposed hierarchical thresholding. Green regions are rejected because of their concavity. Cyan regions are rejected because the DAPI/GFP intensity analysis indicates that cells are probably not in interphase. Only nuclei circled in red remain in our analysis, and for the subsequent analysis, we consider the orange segmentation, obtained after morphological operations: 13x13 dilation, followed by 3x3 erosion.

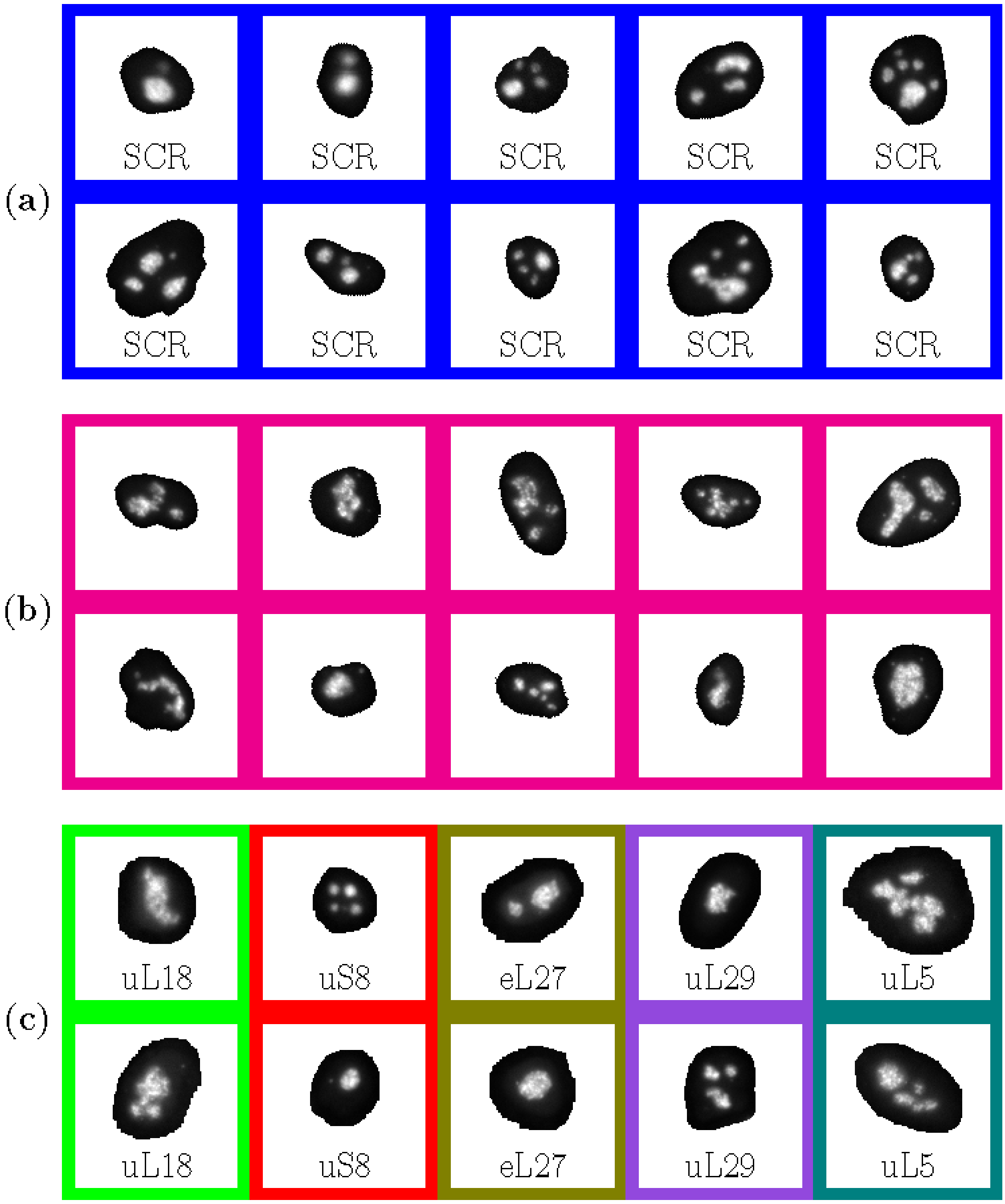

Supplementary Fig. 16: Samples of nucleoli from SCR-treated control cells (**a**), from cells with a range of high level of nucleolar disruption (**b**) and from cells depleted of a specific protein of interest (**c**). Cell nuclei highlighted with the same color-code were treated with the same siRNA. Images were normalized by percentile 99.9%.

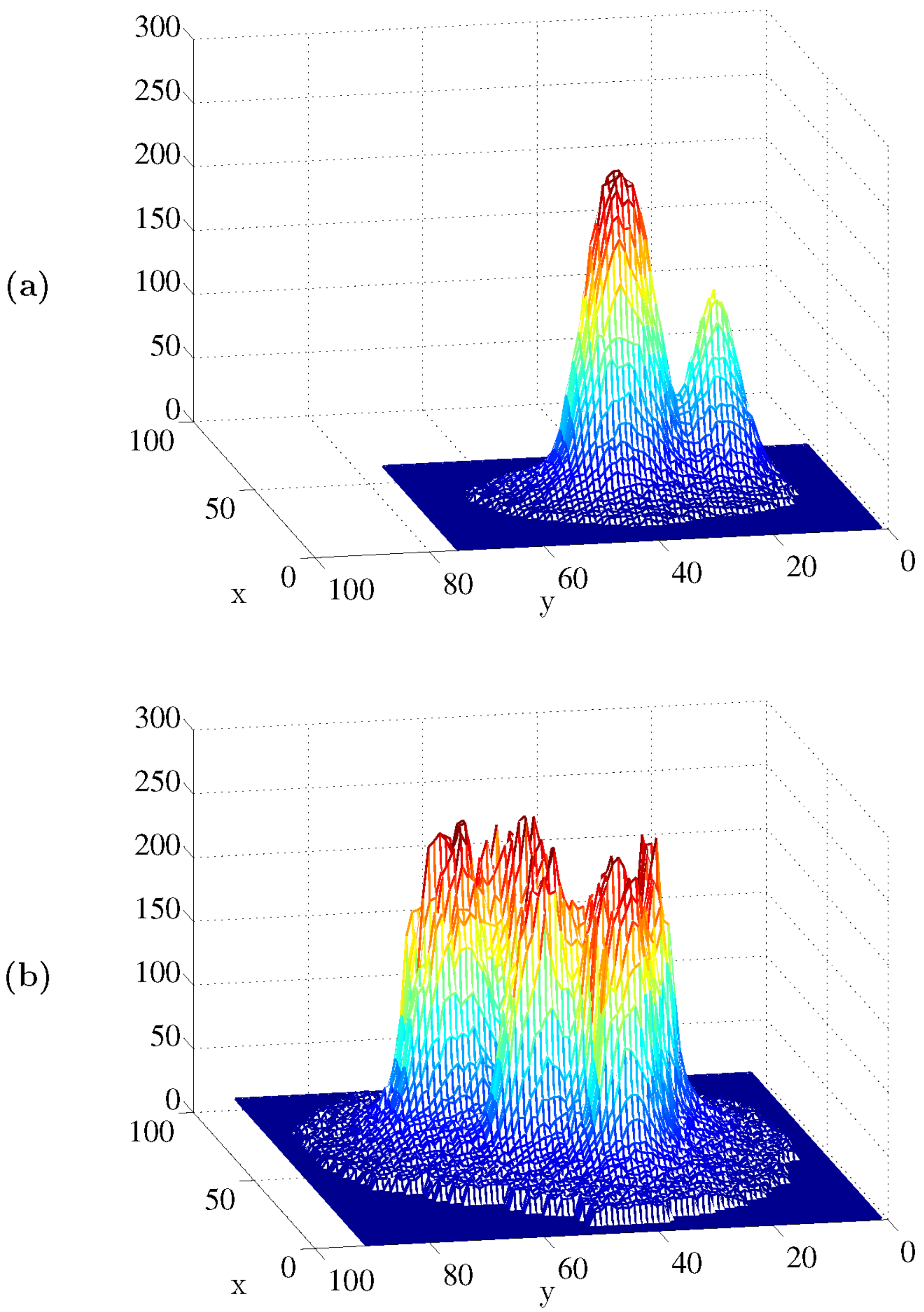

Supplementary Fig. 17: Fibrillarin-GFP intensity profile of nucleoli in a SCR-treated control cell (**a**) or in a cell depleted for the protein uL5 (**b**).

(**a**) corresponds to the nucleus in row 1, column 2 in Supplementary Fig. 16, panel a.

(**b**) corresponds to the nucleus in row 1, column 5 in Supplementary Fig. 16, panel c.

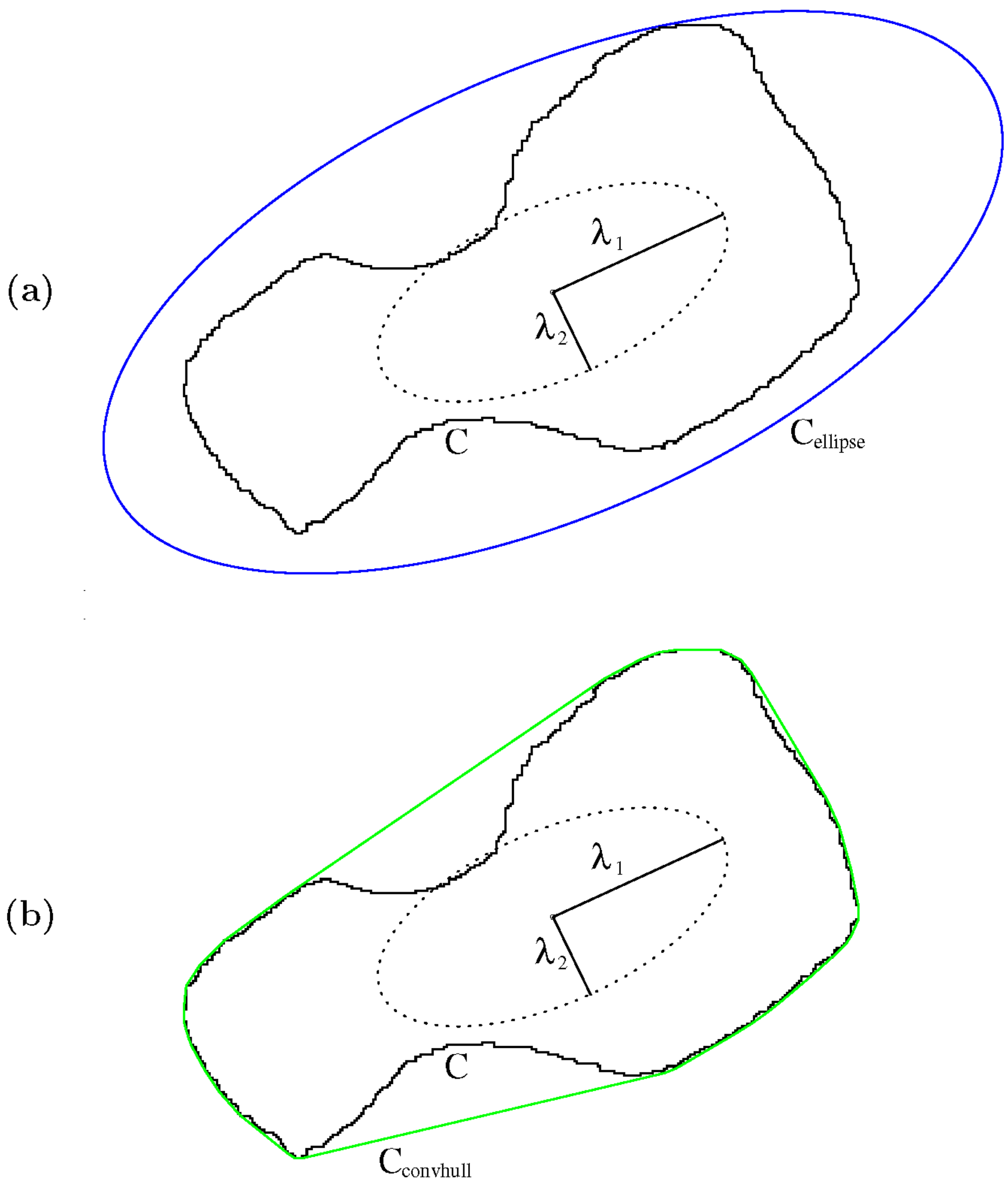

Supplementary Fig. 18: Illustration of the shape factors associated to a connected component *C* (black contour).

(**a**) The elongation factor reflects the ratio between the principal axes second order moments, while the elliptical regularity measures the area ratio of the smallest external ellipsoid (in blue) to the connected component.

(**b**) The concavity ratio measures the area ratio between the convex hull (in green) and the connected component.

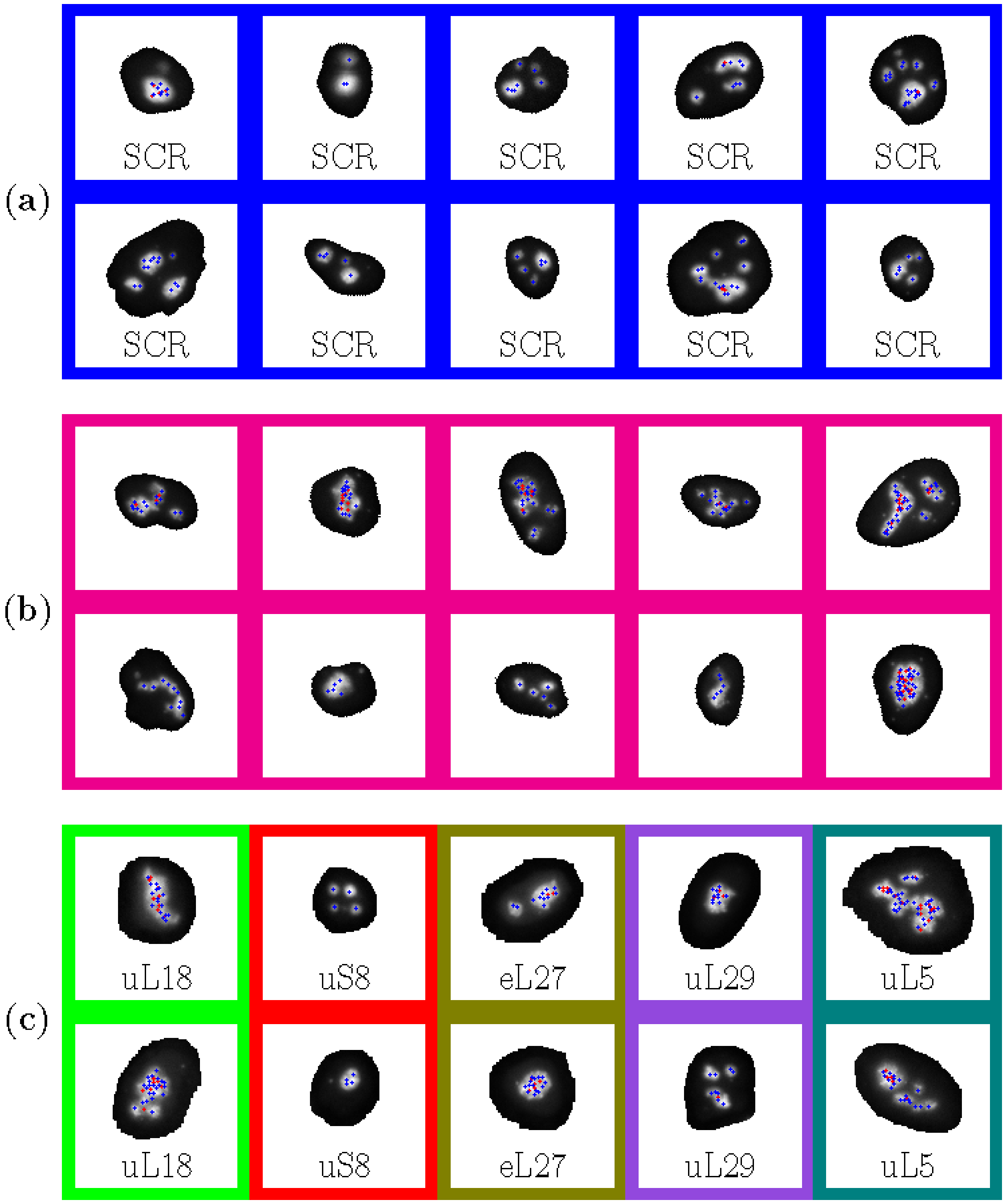

Supplementary Fig. 19: Distribution of local maxima (blue dots) and local minima (red dots) in the images shown in Supplementary Fig. 16. It is apparent that the density of local minima is higher in nucleoli from cells with high level of nucleolar disruption (panel **b**) or from cells depleted of a protein of interest (panel **c**) than in nucleoli from SCR-treated control cells (panel **a**).

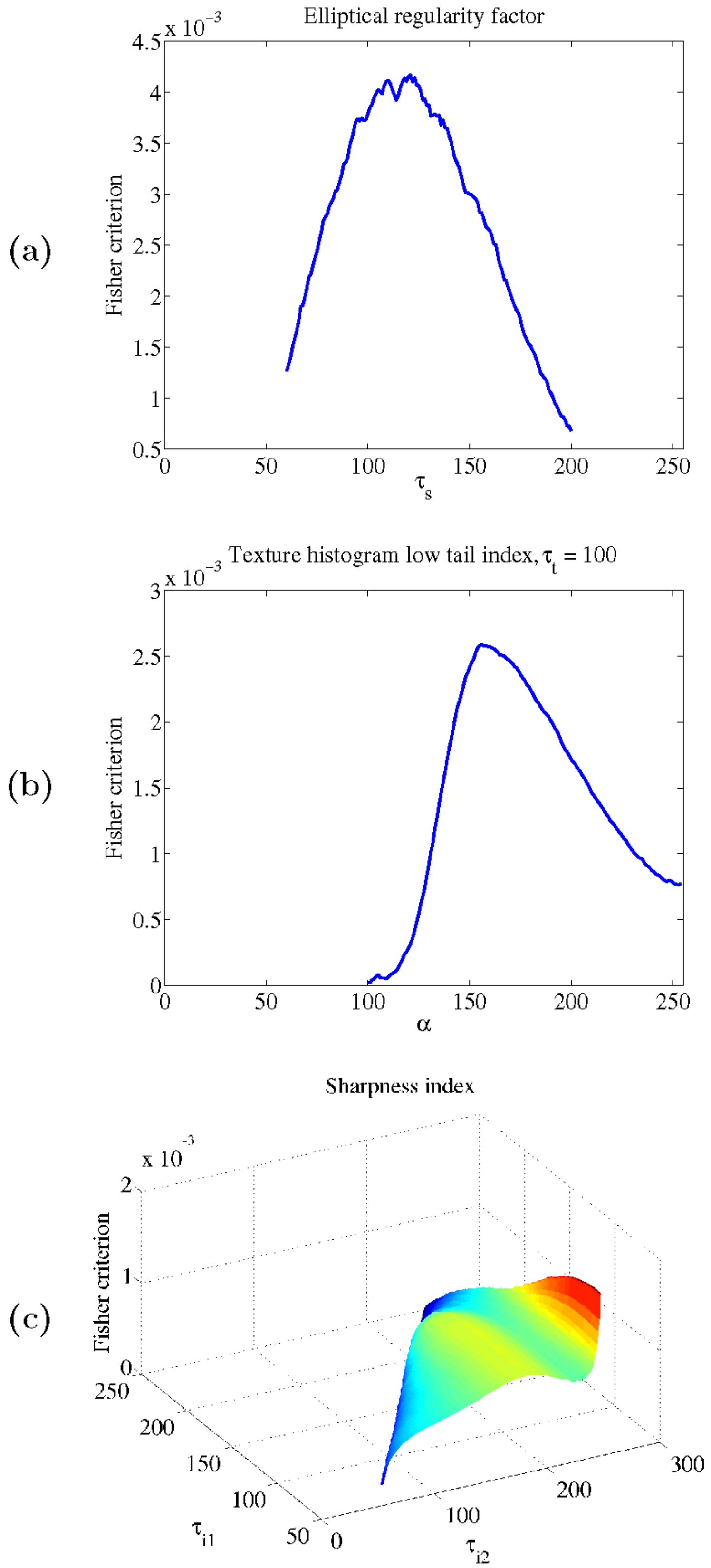

Supplementary Fig. 20: Fisher's optimization criterion as a function of three features (**a**, **b**, and **c**) parameters.

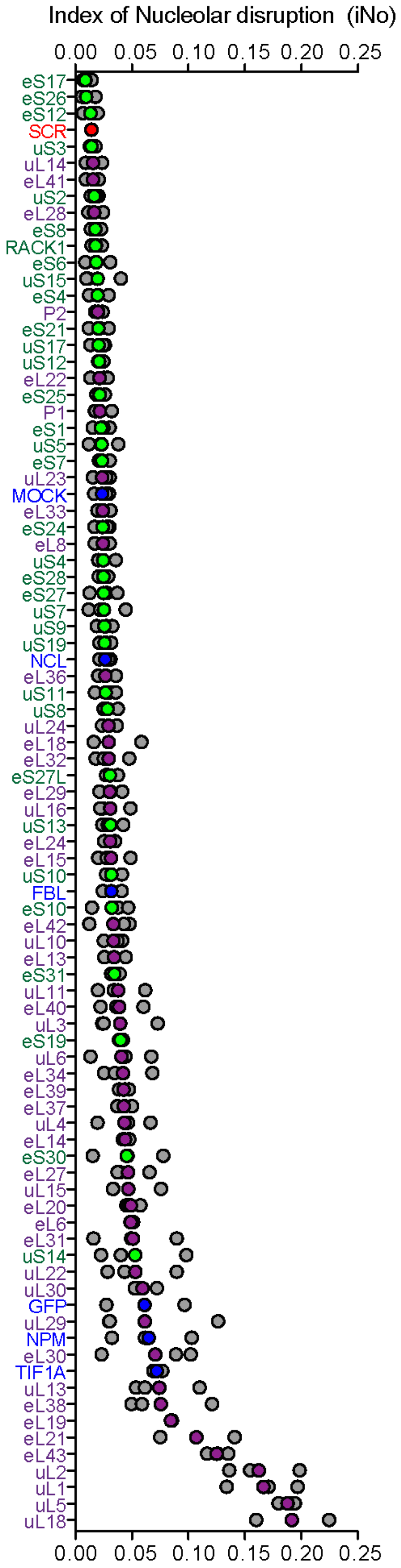

Supplementary Fig. 21: L1-norm of the discrepancy vectors computed from the images presented in our experimental screens of 80 r-proteins (see www.RibosomalProteins.com).

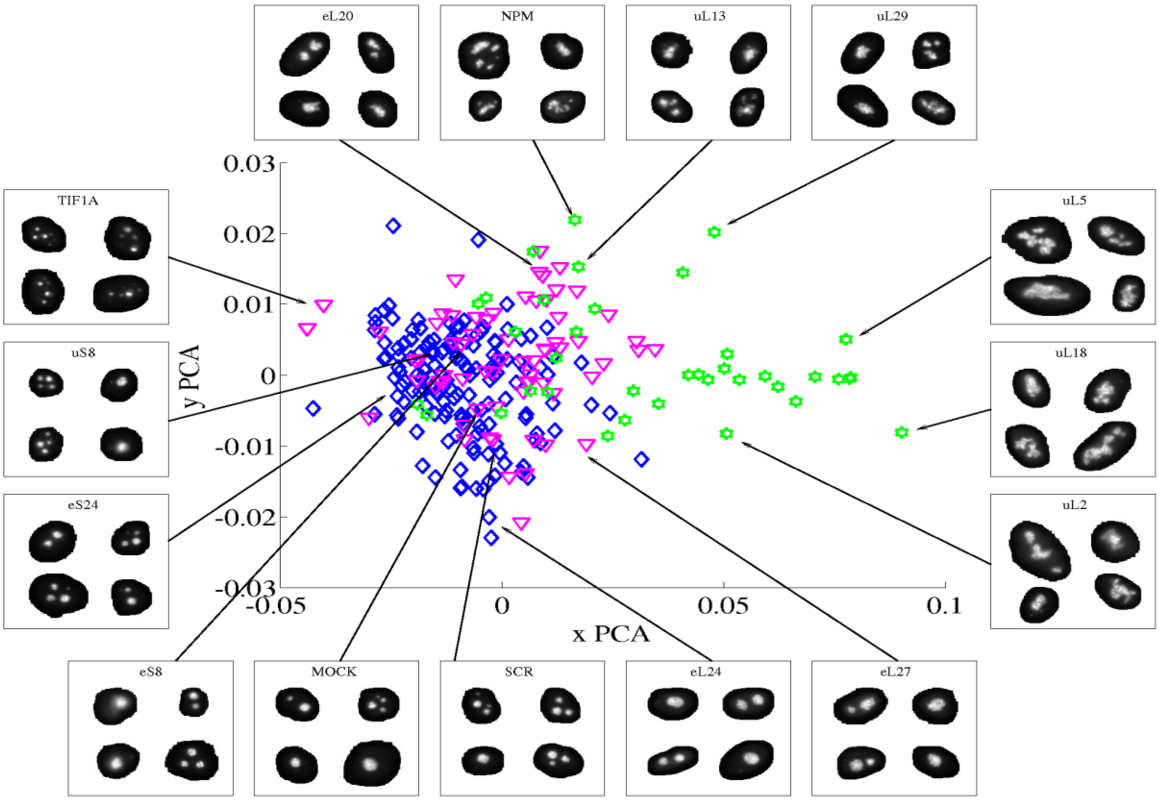

Supplementary Fig. 22: Distribution of the discrepancy vectors according to the two most significant components derived from a PCA analysis.
Color code: blue, no (or weak) impact on nucleolar structure; magenta, intermediate impact on nucleolar structure; green: strong effects on nucleolar structure. Classification on manual ground truth (referred to as “manual classification”, see Supplementary Fig. 2).

**(a)**	**1.22%**<br>**2**<br>**200**	**12.57%**<br>**1**<br>**∞**	**2.83%**<br>**1**<br>**∞**	**1.20%**<br>**1**<br>**216**	**1.40%**<br>**1**<br>**240**
	**6.02%**<br>**2**<br>**∞**	**4.05%**<br>**1**<br>**∞**	**0%**<br>**1**<br>**∞**	**9.12%**<br>**3**<br>**173**	**5.05%**<br>**1**<br>**∞**
**(b)**	**31.51%**<br>**1**<br>**104**	**27.72%**<br>**8**<br>**101**	**51.30%**<br>**5**<br>**112**	**29.93%**<br>**7**<br>**142**	**4.93%**<br>**6**<br>**190**
	**29.58%**<br>**5**<br>**∞**	**11.17%**<br>**2**<br>**∞**	**8.57%**<br>**2**<br>**∞**	**20.83%**<br>**2**<br>**∞**	**7.78%**<br>**7**<br>**140**
**(c)**	**22.42%**<br>**6**<br>**132**	**0%**<br>**1**<br>**∞**	**2.18%**<br>**1**<br>**165**	**6.83%**<br>**3**<br>**162**	**11.21%**<br>**10**<br>**118**
	**22.51%**<br>**5**<br>**111**	**1.77%**<br>**2**<br>**∞**	**4.78%**<br>**4**<br>**159**	**6.17%**<br>**1**<br>**149**	**14.55%**<br>**4**<br>**160**

Supplementary Table 1: Histogram low tail index ($\tau_t = 100$, $\alpha = 150$), number of uplands region ($\tau_{i1} = 100, \tau_{i2} = 200$), and smallest local minimum ($\tau_a = 100$) (∞ indicates the absence of a local minimum) in the largest connected component of the texture segmented images derived from Supplementary Fig. 15.

(a) nucleoli from 12 SCR-treated control cells, (b) nucleoli with high level of nucleolar disruption, (c) nucleoli from cells depleted of a protein of interest (see Supplementary Fig. 15).

**Feature**	**Principal component**	**Second principal component**
AA_{lcc}	0.4808	-0.4864
SR_{lcc}	-0.5503	-0.6191
TH_{lcc}	0.3772	0.3789
TLM_{lcc}	-0.4470	0.2151
TV_{lcc}	0.3521	-0.4363

Supplementary Table 2: Two most significant PCA vectors.

Supplementary Note 1: pseudo-code for nuclei segmentation

```
Inputs:
I: image
λ_1 < λ_2 < ⋯ < λ_k < ⋯ < λ_K: thresholds on intensity
S: minimal area threshold for eligible connected component
S_max: maximal nucleus area
∂I : boundary of the image I (first and last rows and columns)


Output:
C : set of connected components associated to segmented nuclei

I_1 ← I > λ_1
D ← connected_components(I_1)
C_1 ← {d ∈ D | area(d) > S}
For k = 2 ⋯ K
   I_k ← I > λ_k
   D ← connected_components(I_k)
   C_k ← ∅
   For all c ∈ C_{k−1}
      L(c) = {d ∈ D | d ⊂ c}              % connected components in c
      L_S(c) = {d ∈ L(c) | area(d) > S}   % components in L(c) larger than S
      If (((|L(c)| == 1) ∧ (|L_S(c)| == 1) ∧ (mean_intensity(L_S(c)) / mean_intensity(c) > 1.15)) ∨ (|L_S(c)| ≥ 2))
      % one big connected component with important gain in intensity
      % OR at least two big connected components
         C_k ← C_k ∪ L_S(c)
      Else % keep c
         C_k ← C_k ∪ {c}
      End if
   End for all
End for
% Remove the connected components that are too big or that touch the image boundary
C ← {c ∈ C_K | (area(c) < S_max) ∧ is_empty(c ∩ ∂I)}
```

Softwares The image processing code was programmed in MatLab. The 3-D models of ribosomal subunits were generated with Pymol v1.5.0.3, the images for microscopy illustrations produced with ImageJ (http://imagej.nih.gov/ij/), and the graphs generated and analyzed with Prism.

Supplementary Methods

Image processing analysis

In this supplemental section of our manuscript, we describe our procedure to extract qualitative and quantitative morphometric information from nucleoli using a low dimensional feature vector. Our aim was to provide a statistically validated tool to discriminate between populations of normal and altered nucleoli.

Overview

First, we describe how individual cell nuclei are segmented within each image of the database, in order to localize individual cells nucleoli. Then, we present the methodology adopted to derive a small set of shape and textural features that characterize the nucleolar morphology in each individual cell nucleus. Finally, we present a quantitative analysis of the differences observed between the distributions of these features in populations of cells depleted for specific gene products and the ones of a reference population. This leads us to use dimensionality reduction techniques, to stratify and rank the r-proteins according to their impact on the nucleolar structure. The stratification is based on a Principal Components Analysis (PCA) of the five distances (d_k, $k \leq 5$) measured between the distributions observed for a population of cells depleted for a given r-protein and those observed for a reference population of cells (SCR-treated cells), each distribution being associated to a specific shape/texture feature. For the ranking, we introduce an index of nucleolar disruption, or iNo, corresponding to the sum of the five absolute $|d_k|$ values.

1. Cell nuclei segmentation

As an initial step to delimit cell nucleoli from individual cells we segmented the cell nucleus. Each cell has a single nucleus. The nucleoli are specialized subnuclear domains, which, by definition, are all contained within the nucleus. Segmenting the nucleus, which is dense, compact and easy to score, is thus a mean to delimit the cellular volume that contains nucleoli in each individual cell.

Nuclei are stained with the DNA stain DAPI. The nuclei contours are extracted from the DAPI channel (blue) in two steps. Firstly, large connected components of relatively high intensity are identified using an original adaptive thresholding method. Secondly, the connected components that show significant concavity, indicating they likely correspond to aggregated nuclei, are rejected (Supplementary Fig. 14).

Step 1 consists in a stepwise thresholding of the DAPI images such that nuclei corresponding to sufficiently large connected components of pixels lying above an intensity threshold are selected. Although all parameters of samples preparation (cell seeding, transfection procedure, DAPI staining, cell fixation, etc.) and of image capture (illumination, exposure time, etc.) are fully standardized and automatized, we observed an inherent variability in the DAPI signal intensity of individual nuclei. Supplementary Fig. 15 compares two extreme cases of such variability in panels (a) and (b). To address this we adopt a hierarchical thresholding strategy that progressively refines the segmentation by considering a sequence of *K* increasing thresholds, while exploiting prior knowledge about the size range of human cell nuclei. Detailed nuclei segmentation pseudo-code is

provided in Supplementary Note 1. In short, let λ_k denote the kth intensity threshold, with $0 < k \leq K$, and $\lambda_k < \lambda_{k+1}$, $\forall\ k < K$. Let I_k denote the thresholded binary image, i.e. $I_k(\mathbf{x}) = 1$ if $I(\mathbf{x}) > \lambda_k$, and 0 elsewhere, with $\mathbf{x} \in [1,H] \times [1,W]$, H and W denoting the height and width of the image, respectively. We also introduce C_k to denote the set of sufficiently large (compared to a size threshold S) connected components at step k. At initialization, C_1 includes the connected components in image I_1 that are larger than S. The set C_{k+1} of connected components at step $k+1$ is then derived iteratively from C_k and I_{k+1}, as follows. C_{k+1} is initialized to the empty set. For each connected component $c \in C_k$, we considered the list L(c) of connected components in I_{k+1} that are included in c. If L(c) includes at least two connected components with sizes larger than S, then those connected components are added to C_{k+1}. If the list L(c) includes a single connected component c' larger than S, then either c' or c are added to C_{k+1}, depending on whether the gain in mean intensity between c and c' is larger or smaller than 15%, respectively. Note that a higher gain in mean intensity reveals a more accurate segmentation, and that the value of 15% was set empirically. If the list L(c) does not include any component larger than S, then c is added to C_{k+1}. The connected components in C_K that are smaller than a threshold S_{max} are expected to reasonably segment the nuclei. Practically, the threshold S and S_{max} have been set to 500 and 5000 pixels, so as to include most of the size range of human cell nuclei. Regarding λ_k, we considered a sequence of thresholds increasing from 250 to 450 by steps of 20, and from 500 to 1300 by steps of 100. The resulting nuclei segmentation appeared to be relatively independent of the actual sequence used, as long as its range and granularity were sufficient.

As a second step, we consider the rejection of the connected components that likely correspond to multiple nuclei in C_K. For this, we analyzed the convexity of each connected component (Supplementary Fig. 14). Specifically, a number of lines are drawn in parallel to the two principal axis of the connected component. When the connected component is convex, either one or zero segment lies inside the contour, for all parallel lines. In contrast, when the connected component represents aggregated nuclei, it presents a strong concavity and there exist parallel lines that include two or more segments lying inside the contour. If one parallel line supports two sufficiently long inner segments that are separated by a sufficiently long outer segment, the connected component is rejected. In Supplementary Fig. 14, the length of the outer and inner segments respectively correspond to δ_{out}, δ^1_{in}, and δ^2_{in}. Those lengths have to be larger than a threshold of 5 pixels to reject the component. The threshold value has been set empirically to drastically reduce the number of multiple nuclei while keeping most of the single nuclei, compared to a manually generated ground truth. C denotes the subset of C_K that includes all and only all non-rejected components.

Among the nuclei segmented in C, we were only really interested to analyze further those of cells in interphase. To identify them, the DAPI image and the distribution of FIB-GFP (green channel) in the segmented component are considered. A cell is considered to be in interphase if the DAPI is sufficiently dense and spread (if at least 50% of the pixels in the nucleus have a normalized DAPI value larger than 0.47, with the DAPI image being normalized by its maximal value.), and if the FIB-GFP is sufficiently localized (if at least 50% of the pixels in the nucleus have a normalized FIB-GFP value lower than 0.53, with the

FIB-GFP image being normalized by its maximal value). The thresholds were set on the basis of a manual ground truth annotation of the images. These thresholds are quite stringent and their use results in the loss of a small fraction of cells in interphase, however our aim to only consider cells in interphase for further analysis is successfully achieved.

Finally, as a post-processing step, to ascertain all nucleoli of a cell are contained in each segmented regions, we apply basic mathematical morphology to close and enlarge each connected regions. Specifically, a dilation by a 13 x 13 structuring element was followed by a 3 x 3 erosion.

As depicted in Supplementary Fig. 15, our proposed method segments nuclei effectively, both in highly (panel a) and weakly (panel b) contrasted DAPI images. In this figure, the set of connected components that segment interphase nuclei are depicted in red. A reasonable detection rate was achieved, in conjunction with a very small false positive rate. This result is well suited to our needs since we are not interested in detecting all nuclei but rather in collecting a sufficient number of representative nucleoli patterns samples from each FIB-GFP image.

2. Nucleoli image features

This section introduces the image features that we consider to discriminate normal and altered nucleoli morphology in FIB-GFP images[1].

Section 2.1 introduces a number of original parameterized image features to measure the most significant visual differences observed in a set of representative nucleoli. Section 2.2 optimizes the parameters of those features, so as to maximize the discrimination between the distributions of the features in r-proteins-depleted cells and SCR-treated control cells.

2.1 Discriminant nucleolar morphometric features

The distribution of the nucleolar masses within a cell nucleus soon appeared to be subject to important stochastic variability. This is well illustrated in Supplementary Fig. 16, showing digitally resected nucleoli from control cells (panel a) and from cells depleted of specific proteins of interest (panels b, c). In these, the spatial organization of the nucleolar masses with respect to the nucleus center or to its principal axis fluctuates substantially across the images of a given panel, and do not help in differentiating the images from each panel. Hence, any features that would measure how the nucleolar topology is defined in terms of the absolute and normalized position of its components are not relevant to our problem. For example, the popular object recognition approaches that define the object appearance in terms of the intensities and gradients observed on small patches defined by their size and location in a normalized image do not help[12-14]. The same holds true regarding transport-based features[15-17], since they measure the discrepancy compared to a reference distribution of masses, which is not available here.

[1] For each segmented nucleus, its FIB-GFP signal is normalized by a percentile of 99.9% and all the features presented in this section are computed on this normalized signal.

We therefore consider the definition of a set of ad-hoc features that are independent of the actual position of the nucleoli within the nucleus, while still reflecting the spatial spreading of the nucleolar masses. Those features have been defined based on a manually-selected set of image samples, depicting typical normal and abnormal nucleolus patterns (see Supplementary Fig. 16). The manual extraction of representative samples is required to derive features that are relevant to the problem at hand, i.e. to make sure that the set of investigated features are able to discriminate among the variety of nucleolus appearances. However, to avoid (over)fitting our investigated features to those manually annotated samples, in the rest of the section, each feature is systematically defined as a parametric function, so that its parameters can be optimized over the entire database to make the feature can differentiate between normal and gene-depleted cells images (see Section 2.2).

To derive our set of parametric features, Supplementary Fig. 16 presents a set of manually-selected cells nucleoli images that are representative of the appearance diversity in reference control cells, and in cells depleted of proteins of interest. Panel c (resp. b), shows that the nucleoli from cells depleted of specific proteins of interest (resp. nucleoli with very high level of disruption) are generally spread over large and often irregular shapes, which contrasts with the rather circular spot distribution observed in control cells (panel a).

In addition, the 3-D graphs depicted in Supplementary Fig. 17 reveal that the distribution of FIB-GFP intensity of normal nucleoli is smoother and less peaky than in nucleoli of cells depleted of a protein of interest. Those observations motivated us to use features that characterize: (i) the area of support, (ii) the shape regularity, and (iii) the variations of intensities, i.e. the texture, of the nucleolar GFP signal.

Practically, for each nucleus, all our proposed features are defined with respect to the segmentation of the nucleolus masses into a set of disjoint connected components. This segmentation is obtained by FIB-GFP image thresholding, which means that a pixel is considered to be part of the nucleolus if its intensity lies above the threshold. For each feature, the segmentation threshold parameter is defined automatically according to the method proposed in the next section, so as to maximize the separation between the distributions of the features for SCR-treated control cells and for cells depleted of proteins of interest. Hence, the segmentation threshold is a feature parameter, and might vary from one feature to the other. Other feature parameters are optimized similarly, and are thus defined automatically, as described in the next section.

-Area of support:
To characterize the area of support of the nucleolus, we first consider the size and number of connected components obtained after thresholding with a so-called area segmentation threshold τ_a. Specifically,

- AA_{lcc} measures the area of the largest connected component in the thresholded image, and
- AN_{cc} denotes the number of connected components in the nucleus.

In addition, to characterize the sharpness of the intensity gradient along the frontier delimiting the nucleolar masses, we introduced a sharpness index *AS* that measures the ratio of the nucleus pixels that respectively lies above two thresholds τ_{i1} and τ_{i2}, with $\tau_{i1} > \tau_{i2}$.

-Shape and texture:
To characterize the shape and the texture of the nucleolus, we only consider the largest connected component obtained after segmentation, because the small-sized components naturally tend to reduce to single circular peaks, making the largest connected component more representative with respect to shape and texture.

<u>Shape:</u>
To quantify the nucleolus shape regularity, we adopt a shape segmentation threshold τ_s, and consider three distinct shape factors to characterize the shape of the largest connected component in the segmented image. Each factor describes the shape independently of its size.

They are illustrated in Supplementary Fig. 18, and correspond to:

- The elongation shape factor SE_{lcc}, which is defined as the square root of the ratio of the two second order moments, λ_1 and λ_2 , of the connected component *c* around its principal axes;
- The elliptical regularity factor SR_{lcc}, which is defined as the ratio between the area of the connected component, and the area of the smallest ellipse lying outside the connected component, and having the same center, the same principal axes, and the same elongation than the connected component.
- The concavity factor SC_{lcc}, which is defined as the ratio between the area of the connected component and the area of its convex hull.

<u>Texture:</u>
To characterize the nucleolar texture pattern, after having investigated without any success (data not shown) some conventional texture descriptors such as the local binary patterns[18], the region covariance[19], or the grey level aura matrices[20], we introduced a number of original scalar metrics to reflect the distribution of intensities inside the largest connected component segmented based on a texture segmentation threshold τ_t. Those metrics are:

- The texture histogram low tail index TH_{lcc} , which measures the percentage of pixels that lie below some intensity threshold α, while being located inside the erosion of the connected component by a 3 x 3 pixels structuring element[21]. An erosion is applied to the shape to get rid of the low intensity pixels lying on the border of the shape;
- The texture uplands index TU_{lcc}, which is defined to be the number of connected regions lying above a threshold β, while being inside the connected component;
- The texture peaks index TP_{lcc}, which is defined to be the number of local maxima in the connected component;
- The texture valleys index TV_{lcc} , which is defined to be the number of local minima in the connected component;

- The texture local minimum TLM_{lcc}, which is defined as the intensity of the smallest local minimum in the connected component;

As can be observed in Supplementary Table 1 and Supplementary Fig. 19, those scalar features have reasonably distinct values for the representative images depicted in Supplementary Fig. 16. Supplementary Fig. 19 illustrates that the number of local minima (red dots) is generally more important in morphologically disrupted nucleoli (panel b and c) than in control nucleoli (panel a). We thus expect that they are appropriate to differentiate normal from altered nucleoli (see below).

2.2. Supervised optimization of features parameters

This section explains how the parameters involved in the definition of the above features are selected to best discriminate between normal and altered nucleoli.

Following the Fisher's criterion introduced by the popular Linear Discriminant Analysis (LDA)[22], we propose to select those parameters so as to maximize the separation between the features distributions that we want to discriminate.

In short, the Fisher's optimization criterion considers the problem of estimating whether a feature can discriminate between two classes of data, knowing the feature values for a representative set of data samples from each class. A natural step to answer this question consists in looking at the average (or the mean) of the feature values from each class. Intuitively, the closer the means are, the less discriminant the feature is. This is because a large distance between the means implies that the gap between the classes is expected to be large in the corresponding feature space. However, before drawing a conclusion about this gap, we also need to account for the spreading of the features around their respective mean, so that we can decide whether a given distance between the means is significant or not. Based on this reasoning, Fisher has defined the separation between the distributions associated to two classes of observations to be the ratio of the squared distance between the means to the sum of the variance within each class[22]. We adopted the same criterion to optimize the parameters of our features. In other words, a discriminant feature is one for which the class-means are well separated, measured relative to the (sum of the) variances of the data assigned to a particular class.

Formally, considering a feature, parameterized by a vector ***p*** lying in a parameter space *P*, we assume that the feature distributions are known as a function of ***p***, for the two classes of observations that we want to best discriminate. Then, the Fishers' optimization criterion informs us that the vector ***p**** that maximizes the separation between the class distributions is defined as:

$$\boldsymbol{p}^* = \underset{\boldsymbol{p} \in P}{\operatorname{argmax}} \frac{[\mu_1(\boldsymbol{p}) - \mu_2(\boldsymbol{p})]^2}{\sigma_1(\boldsymbol{p})^2 + \sigma_2(\boldsymbol{p})^2} \quad ,$$

where $\mu_1(\boldsymbol{p})$, $\mu_2(\boldsymbol{p})$ and $\sigma_1(\boldsymbol{p})$, $\sigma_2(\boldsymbol{p})$ respectively denote the means and standard deviations of the distributions of the feature of interest, measured with parameter ***p*** for the two classes. This Fisher's criterion is equivalent to the Welch's adaptation of the t-test[23], widely used in image-based morphometry[24].

Since it relies on the distributions of features that are observed for the two classes to discriminate, our proposed parameter optimization method has to be supervised. In our case, we know by design of our experimental set up which cells images correspond to control and (gene-depleted) test cells. Hence, we can readily identify pairs of distributions that should be discriminated one from the other. Practically, we selected the parameters so as to maximize the sum of the separation measured between each pair of distributions extracted from SCR-treated control cells, and from cells depleted of a protein of interest.

Supplementary Fig. 20 presents the Fisher's optimization criterion for three different features, as a function of their associated parameter. We observe that the parameter selection significantly impact the discriminative power of the feature.

It is worth noting here that our methodology has been defined to limit the impact of supervision on the outcome of the data mining process presented in Section 3. Specifically, supervision is deliberately restricted to the independent selection of individual feature parameters, without being involved in how the resulting features will be combined in the next section, based on a strictly unsupervised approach. Moreover, by defining the features parameters to differentiate distributions of samples extracted from the same culture, we avoid biasing the selection of features induced by the exploitation of a class containing different kind of deviations compared to the reference class.

3. Nucleolar features distribution analysis

This section analyzes how the distributions of the features of nucleoli observed in SCR-treated control cells compare to those of nucleoli of cells depleted of a protein of interest. As a primary objective, we aimed at quantifying the degree of nucleolar disruption associated with the depletion of a specific protein, based on the analysis of the distribution of the features of the associated nucleoli. Therefore, we introduced a discrepancy vector. Each component of this vector is associated to a specific feature, and measures the separation between the reference distribution and the gene-depleted distribution of interest. We then defined the index of nucleolar disruption, or iNo*,* to be the L1-norm of the discrepancy vector. *This allowed us to rank the degree of severity of nucleolar disruption.*

As a second and complementary outcome, we analyzed the principal components among the set of discrepancy vectors, assuming linear embedding for dimensionality reduction. It allows for extracting and visualizing the major trends affecting the morphology of the nucleolus when it is subject to gene-depletion. *This allowed us to regroup nucleolar disruption phenotypes in classes in an unsupervised fashion.*

3.1. Discrepancy-based distribution characterization

A discrepancy vector is defined so as to summarize how the features distributions associated to a set of nucleoli differ from their corresponding reference distributions.

Formally, let S_r and S_k denote two sets of nucleoli images, respectively obtained from normal reference cells and from cells that have been subject to the kth gene depletion process, i.e. to the kth silencer. For a given image feature f, we define the discrepancy d_k between set S_k and the reference S_r to be the ratio of the difference of the mean feature values on each set to the sum of their variance. The definition naturally extends to N features f_i, $0<i\leq N$, and the ith component of the discrepancy vector $\boldsymbol{d_k}$ associated to the set S_k writes

$$\boldsymbol{d}_k(i) = \frac{\mu_k(i)-\mu_r(i)}{\sqrt{\sigma_k^2(i)+\sigma_r^2(i)}} \ ,$$

with $\mu_k(i)$, $\mu_r(i)$ and $\sigma_k(i)$, $\sigma_r(i)$ denoting the means and standard deviations of the ith feature over sets S_k and S_r, respectively.

3.2. L1 norm of discrepancy vectors

To quantify the disruption level associated with the depletion of a specific protein of interest, we defined an index of nucleolar disruption, or iNo, as the L1-norm of the discrepancy vector computed over the set of nucleoli images obtained from cells depleted for that given protein of interest (Supplementary Fig. 21).

Letting S_k denote the set of nucleoli obtained upon treatment with silencer k, and N be the 11 features defined in Section 2.1, the nucleolus disruption index Δ_k is measured as:

$$\Delta_k = \sum_{i=1}^{N} |d_k(i)|.$$

3.3. Principal component analysis of discrepancy vectors

Principal components analysis (PCA) is an unsupervised method for dimensionality reduction. PCA is used to visualize the most important phenotypic classes observed in our work. PCA searches for directions in the data that have the largest variance, and subsequently projects the data onto it. Following such an approach, we obtained a lower dimensional representation of the data, which removes some of the ‘noisy’, supposedly less meaningful, directions.

Since we are interested to score the nucleolar disruption associated with the depletion of particular proteins of interest, we applied the PCA to the discrepancy vectors that capture the average trends associated with nucleoli in cells depleted with a specific silencer, and not to individual nucleolar feature vectors.

To facilitate the interpretation of the eigenvectors associated to the principal components, we only consider the 5 features that have the largest Fisher’s score, i.e. which best discriminates normal and altered nucleoli. Those features are listed in the first column of Supplementary Table 2.

Supplementary Fig. 22 presents a PCA scatter plot depicting the 2-D points obtained by projecting each discrepancy vector on the two most significant PCA components. We observed that the PCA analysis has successfully found linear combinations of the proposed features that separate out the ground truth clusters, corresponding to different levels of disruption and phenotypic classes. We indeed observe visually that the nature of the disruption changes depending on the position in the scatter plot.

Supplementary Table 2 presents the first two principal components, i.e. the two directions of maximal variability of the projected discrepancy vectors. It reveals the main trends in the disruption process.

From the signs of the components in the first vector, we learned that the dominant disruption process increases the area of the nucleolus (AA_{lcc}), and reduces its elliptical regularity (SR_{lcc}). It also increases the number of low intensity pixels in the segmented nucleoli (TH_{lcc}), as well as the deepness (TLM_{lcc}) and number of local minima (TV_{lcc}), which reflects the scattered nature of the nucleoli spread.

The second vector induces an opposite trend compared to the one induced by the first vector, except for the elliptical regularity (SR_{lcc}) and for the histogram low tail index (TH_{lcc}). Hence, a positive second PCA coefficient tends to foster the decrease of elliptical regularity, while a negative second PCA coefficient mitigates it, compared to what would result from the single vector only. This is reflected in Supplementary Fig. 22 by more regular and circular shapes of the nucleolus masses in case of negative second PCA coefficient. In contrast, a positive second PCA coefficient corresponds to a more severe disruption, with less regular shape than the one observed for nucleoli of similar size in absence of second PCA component.

It is well established in the literature that inhibition of RNA polymerase I (Pol I) leads to a very specific nucleolar morphology alterations referred to as “nucleolar segregation” or “nucleolar caps” [25]. Such caps are for example observed when cells are treated with low doses of actinomycin D, or in our experimental set up, in cells depleted of the Pol I transcription factor TIF1A (Supplementary Fig. 22, caps are seen as tiny bright dots).

As an illustration that the PCA analysis is a powerful method to classify in an unsupervised fashion distinct nucleolar disruption phenotypes, nucleoli of cells depleted of TIF1A, which in agreement with the literature[25] have a markedly different nucleolar disruption phenotype by comparison to the other control or test cells, correspond to two magenta triangles totally isolated in the upper left corner of the graph and characterized by a negative first PCA component value, which is in contrast to most other magenta triangles corresponding to cells depleted for other test genes.

Supplementary References

1. Tafforeau, L. *et al.* The complexity of human ribosome biogenesis revealed by systematic nucleolar screening of Pre-rRNA processing factors. *Molecular cell* **51**, 539-551 (2013).
2. O'Donohue, M.F., Choesmel, V., Faubladier, M., Fichant, G. & Gleizes, P.E. Functional dichotomy of ribosomal proteins during the synthesis of mammalian 40S ribosomal subunits. *The Journal of cell biology* **190**, 853-866 (2010).
3. Robledo, S. *et al.* The role of human ribosomal proteins in the maturation of rRNA and ribosome production. *Rna* **14**, 1918-1929 (2008).
4. Chen, S.S. & Williamson, J.R. Characterization of the ribosome biogenesis landscape in E. coli using quantitative mass spectrometry. *Journal of molecular biology* **425**, 767-779 (2013).
5. Sykes, M.T. & Williamson, J.R. A complex assembly landscape for the 30S ribosomal subunit. *Annual review of biophysics* **38**, 197-215 (2009).
6. Talkington, M.W., Siuzdak, G. & Williamson, J.R. An assembly landscape for the 30S ribosomal subunit. *Nature* **438**, 628-632 (2005).
7. Mulder, A.M. *et al.* Visualizing ribosome biogenesis: parallel assembly pathways for the 30S subunit. *Science* **330**, 673-677 (2010).
8. Jomaa, A. *et al.* Functional domains of the 50S subunit mature late in the assembly process. *Nucleic acids research* **42**, 3419-3435 (2014).
9. Ferreira-Cerca, S. *et al.* Analysis of the in vivo assembly pathway of eukaryotic 40S ribosomal proteins. *Molecular cell* **28**, 446-457 (2007).
10. Ferreira-Cerca, S., Poll, G., Gleizes, P.E., Tschochner, H. & Milkereit, P. Roles of eukaryotic ribosomal proteins in maturation and transport of pre-18S rRNA and ribosome function. *Molecular cell* **20**, 263-275 (2005).
11. Gamalinda, M. *et al.* A hierarchical model for assembly of eukaryotic 60S ribosomal subunit domains. *Genes & development* **28**, 198-210 (2014).
12. Viola, P. & Jones, M. Robust real-time object detection. *IJCV* **57**, 137-154 (2004).
13. Dalal, N. & Triggs, B. Histograms of oriented gradients for human detection. *IEEE International Conference on Computer Vision and Pattern Recognition* (2005).
14. Felzenszwalb, P., Girshick, R.B., McAllester, D. & Ramanan, D. Object detection with discriminatively trained part-based models. *IEEE Trans on PAMI* **32**, 1627-1645 (2010).
15. Gangbo, W. & McCann, R. The geometry of optimal transportation. *Acta Math* **177**, 113-161 (1996).
16. Haker, S., Angenent, S. & Tannenbaum, A. Minimizing flows for the monge-kantorovich problem. *SIAM J. Math Analysis* **35** (2003).
17. Wang, W., Slepcev, D., Basu, S., Ozolek, J.A. & Rohde, G.K. A linear optimal transportation framework for quantifying and visualizing variations in sets of images. *Int. J. Computer Vision* **101**, 254-269 (2013).

18. Ojala, T., Pietikäinen, M. & Harwood, D. A comparative study of texture measures with classification based on feature distributions. *Pattern Recognition* **29**, 51-59 (1996).
19. Tuzel, O., Porikli, F. & Meer, P. Region covariance: a fast descriptor for detection and classification. *ECCV* (2006).
20. Qin, X. & Yang, Y.-H. Similarity measure and learning with gray level aura matrices (GLAM) for texture image retrieval. *CVPR* (2004).
21. Serra, J. *Image analysis and mathematical morphology*. (1982).
22. Fisher, R.A. The use of multiple measurmements in taxonomic problems. *Annals of Eugenics* **7**, 179-188 (1936).
23. Welch, B.L. The generalization of "Student's" problem when several different population variances are involved. *Biometrika* **34**, 28-35 (1947).
24. Wolfe, P. *et al.* Using nuclear morphometry to discriminate the tumorigenic potential of cells: a comparison of statistical methods. *Cancer Epidmiol Biomarkers Prev* **13**, 976-988 (2004).
25. Hernandez-Verdun, D., Roussel, P., Thiry, M., Sirri, V. & Lafontaine, D.L.J. The nucleolus: structure/function relationship in RNA metabolism. *Wiley interdisciplinary reviews. RNA* **1**, 415-431 (2010).
